# HetFCM: functional co-module discovery by heterogeneous network co-clustering

**DOI:** 10.1093/nar/gkad1174

**Published:** 2023-12-13

**Authors:** Haojiang Tan, Maozu Guo, Jian Chen, Jun Wang, Guoxian Yu

**Affiliations:** School of Software, Shandong University, Jinan 250101, Shandong, China; Joint SDU-NTU Centre for Artificial Intelligence Research, Shandong University, Jinan 250101, Shandong, China; College of Electrical and Information Engineering, Beijing Uni. of Civil Eng. and Arch., Beijing 100044, China; College of Agronomy & Biotechnolog, China Agricultural University, Beijing 100193, China; Joint SDU-NTU Centre for Artificial Intelligence Research, Shandong University, Jinan 250101, Shandong, China; School of Software, Shandong University, Jinan 250101, Shandong, China; Joint SDU-NTU Centre for Artificial Intelligence Research, Shandong University, Jinan 250101, Shandong, China

## Abstract

Functional molecular module (i.e., gene–miRNA co-modules and gene–miRNA–lncRNA triple-layer modules) analysis can dissect complex regulations underlying etiology or phenotypes. However, current module detection methods lack an appropriate usage and effective model of multi-omics data and cross-layer regulations of heterogeneous molecules, causing the loss of critical genetic information and corrupting the detection performance. In this study, we propose a heterogeneous network co-clustering framework (HetFCM) to detect functional co-modules. HetFCM introduces an attributed heterogeneous network to jointly model interplays and multi-type attributes of different molecules, and applies multiple variational graph autoencoders on the network to generate cross-layer association matrices, then it performs adaptive weighted co-clustering on association matrices and attribute data to identify co-modules of heterogeneous molecules. Empirical study on Human and Maize datasets reveals that HetFCM can find out co-modules characterized with denser topology and more significant functions, which are associated with human breast cancer (subtypes) and maize phenotypes (i.e., lipid storage, drought tolerance and oil content). HetFCM is a useful tool to detect co-modules and can be applied to multi-layer functional modules, yielding novel insights for analyzing molecular mechanisms. We also developed a user-friendly module detection and analysis tool and shared it at http://www.sdu-idea.cn/FMDTool.

## Graphical Abstract

**Graphical Abstract:**
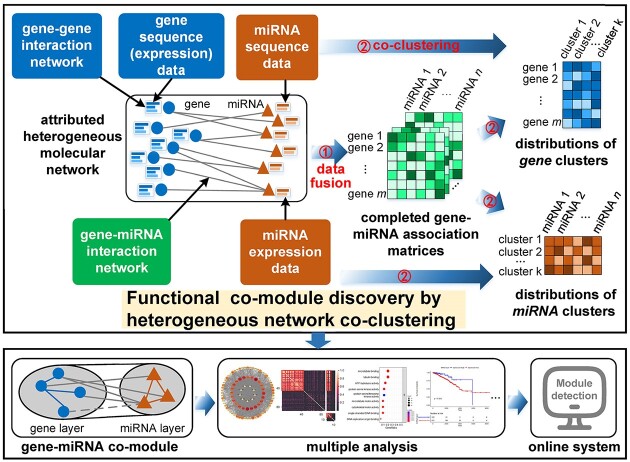

## Introduction

With the development of high-throughput techniques and the rapid accumulation of multi-omics data, it is recognized that the functions and phenotypes of living organisms carry out in a modular manner (1,2). For example, in clear cell renal cell carcinoma, a chromatin remodeling module composed with a number of mutated genes is detected and it engages with a wide variety of processes, such as regulation of hormone receptors and immune-related signaling (3). Therefore, it is essential to develop a functional module detection tool rather than annotating functions of individual molecules, which can uncover the complex regulatory mechanisms for important pathology or phenotypes, consequently paving the way for the disease treatment, plant breeding and many others (4–6).

Some computational methods (7,8) have been proposed to detect functional modules from biological omics data. These methods can be roughly divided into supervised and unsupervised ones. Supervised methods (9–11) usually train a classifier based on the characteristics of known modules to infer new modules. Unfortunately, the quality and quantity of known modules can not be guaranteed. Unsupervised methods (12,13) directly detect functional modules through molecular network topology or attributes. However, these methods are often limited to single-layer modules with only one type of molecules (i.e. genes or miRNAs), and neglect the complex regulations among different types of molecules.

Compared with single-layer modules, co-modules (also called bilayer modules) with two types of molecules, are particularly important to reveal the molecular heterogeneity in the module and elucidate complex regulations, which more precisely uncover genetic mechanism of modules associated with phenotypes (14–16). Besides, triple-layer (or even-higher order) modules with ≥3 types of molecules can be also found by the shared molecules between co-modules. Therefore, co-module detection is paramount important and has attracted increasing attention (4,17).

Co-module detection is canonically implemented by clustering-based methods (6), which proceed on the assumption that molecules with similar features (i.e., expression profiles) or interactions usually have similar functions and tend to be clustered into the same module. Based on the detection techniques, existing methods can be divided into three classes: enumeration, independent clustering and collaborative clustering. The *enumeration*-based methods detect highly connected sub-graphs in biological networks as co-modules through iterative strategy (18–20). Although these enumeration methods have good interpretability, they typically rely on the hyperparameters and are sensitive to the putative network interaction weights. The *independent clustering*-based methods first cluster a type of molecules, and then add another type of molecules or molecular clusters to the existing cluster based on the associations (interactions) between two types of molecules (21). These methods only consider the known molecular associations in clustering, and may lack a good account of molecular attributes and potential regulations between different molecules. The *collaborative clustering*-based methods (4,17,22,23) can group two types of molecules simultaneously, they aim to extract the shared patterns of heterogeneous molecules and find out co-modules. Although these methods make good achievements for function module detection, they still have some limitations. First, they mainly focus on transcriptomic data and are difficult to extend to other omics data (such as genomic sequence data and protein-protein interaction data), suffering the loss of important genetic information for deciphering molecular mechanisms. Second, they usually adopt network constraints to integrate molecular omics data and network topology, consequently failing to capture the potential cross-layer associations, which help the detection of co-modules. Furthermore, they lack expandability and cannot differentially integrate cross-talks and multi-omics data of heterogeneous molecules, compromising the detection of co-modules.

**Figure 1.**
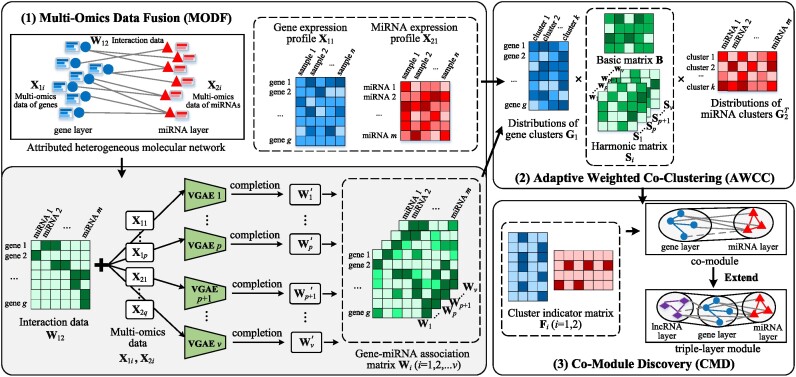
The overall framework of HetFCM, which mainly contains three parts: (1) multi-omics data fusion (MODF) applies multiple VGAE to obtain a series of gene–miRNA association matrices; (2) adaptive weighted co-clustering (AWCC) gains the molecular cluster distributions by adeptly fusing association data matrices and expression data; (3) co-module discovery (CMD) detects molecular co-modules based on cluster distributions and thresholds.

To this end, we propose HetFCM to detect functional co-modules on heterogeneous molecular network by co-clustering, as depicted in Figure 1. Experimental results on Human and Maize datasets show that the co-modules detected by HetFCM exhibit more compact structures and enriched functions than six competitive methods and its degenerated variants. On Human dataset, HetFCM identified 30 novel gene–miRNA co-modules. Among them, gene layer modules and miRNA layer modules in 22 co-modules (73%) jointly perform the same cellular function or participate in the same pathway, which can help to explain how the interplays of genes and miRNAs collaboratively impact cancers. HetFCM can also identify the co-modules associated with cancer subtypes. On Maize dataset, several functional modules are detected and significantly associated with important agronomic traits, such as lipid storage, drought tolerance and oil content, which hold particular importance for maize breeding. These detected co-modules include the potential cooperative driver pathways and gene–miRNA interactions, which give new insights on molecular mechanisms of etiology or phenotypes. Moreover, HetFCM can be extended to identify multi-layer (i.e. gene–miRNA–lncRNA) functional modules, and decipher more intricate genetic mechanisms.

## Materials and methods

### Datasets and data preprocessing

MicroRNAs (miRNAs) are a category of short non-coding RNAs that play crucial roles in diverse regulatory processes, including cell differentiation, proliferation, apoptosis and tumorigenesis (24). miRNAs collaborate with genes to constitute functional modules and perform specific functions (4). In this study, we take the gene–miRNA co-module detection as an example to explore the functional co-module discovery algorithm. HetFCM integrates molecular interaction data and multi-omics data, such as molecular expression, nucleotide sequence and interaction data to realize gene–miRNA co-module detection. We apply HetFCM on the Human (1,089 samples) and Maize (36 samples) datasets. The details of these datasets are listed in Supplementary Table S1 in Section 1 of the Supplementary Materials. HetFCM constructs an attributed heterogeneous molecular network based on these data, which can be organized into three forms:

- **Gene–miRNA association data matrix** ${\mathbf {W}_{12}} \in {\mathbb {R}^{g \times m}}$ stores the known interactions between *g* genes and *m* miRNAs. If the *i*-th gene and *j*-th miRNA have a known interaction, **W**_12_(*i*, *j*) = 1; otherwise, **W**_12_(*i*, *j*) = 0. Specifically, **W**_12_ contains 60,900 and 4,291 interactions on Human and Maize datasets, respectively.
- **Gene attribute data matrices** store the multi-omics data of genes. We first download gene expression data of human and maize, where the Human dataset is related with breast cancer while the Maize data is collected at different developmental stages of seed embryos by RNA-sequencing. On Human dataset, we conduct differential expression analysis for genes using the **DESeq2** package (25) in **R** with adjusted *p*-value < 0.01, and |log_2_(fold change)| > 1 to pre-filter genes less related to breast cancer and perform log_2_-transformed on them. On Maize dataset, we retain the expression data of all genes to keep more molecular information due to the sparsity of the gene–miRNA interaction network. At last, we gain the expression profile data of *g* genes across *n* samples, which is represented by expression matrix ${{{\bf X}}_{11}} \in \mathbb {R}{^{g \times n}}$. Besides, we download gene-gene interaction data denoted by ${{{\bf X}}_{12}} \in \mathbb {R}{^{g \times g}}$. We also download gene sequence data and use a 4-mer conjoint triad to convert them into numerical features and denote it by ${{{\bf X}}_{13}} \in \mathbb {R}{^{g \times 256}}$. $\lbrace {\mathbf {X}_{1i}}\rbrace ^p_{i=1} \in {\mathbb {R}^{g \times d_{1i}}}$ store the multi-type attribute data of genes. *d*_1*i*_ is the number of attributes and *p* is the number of data types.
- **MiRNA attribute data matrices** store the multi-omics data of miRNAs. We first download the miRNA expression data of human and maize. On Human dataset, we filter the miRNAs whose expression values are zeros across all of samples and perform log_2_-transformed on them. On Maize dataset, we also retain the expression data of all miRNAs to keep more molecular information. At last, we gain the expression profile data of *m* miRNAs across *n* samples, which is represented by ${{{\bf X}}_{21}} \in \mathbb {R}{^{m \times n}}$. We also download miRNA sequence data and use a 4-mer conjoint triad to convert them into numerical features and denote it by ${{{\bf X}}_{22}} \in \mathbb {R}{^{m \times 256}}$. $\lbrace {\mathbf {X}_{2i}}\rbrace ^q_{i=1} \in {\mathbb {R}^{m \times d_{2i}}}$ denote the *i*-th type of attribute data matrix for miRNAs. *d*_2*i*_ is the number of attributes and *q* is the number of miRNA data types.

### HetFCM

Figure 1 depicts the framework of HetFCM, which consists of three parts, Multi-Omics Data Fusion (MODF), Adaptive Weighted Co-Clustering (AWCC) and Co-Module Discovery (CMD). In MODF, HetFCM introduces multiple Variational Graph Auto-Encoders (VGAE) (26) to integrate association data and multi-omics attributes of heterogeneous molecules, and obtains a series of gene–miRNA association matrices, which help to mine the complex nonlinear interplays between molecules. In AWCC, HetFCM performs adaptive weighted co-clustering to adeptly fuse multiple gene–miRNA association matrices and molecular expression data to obtain the distributions of gene clusters and miRNA clusters. Finally, it leverages the distributions of clusters to discover co-modules. The technical procedure is presented below.

### Step 1: multi-omics data fusion

Gene–miRNA interaction data correspond to a sparse network, while genes and miRNAs have rich attribute data. It is promising to fuse these data to enrich the gene–miRNA associations and thus to boost the detection of gene–miRNA co-modules. VGAE (26) is a self-supervision learning method for network data, and it can use node attributes to reconstruct and complement the sparse associations between nodes (27). Our attributed heterogeneous network contains a variety of molecular node attributes. Therefore, we utilize multiple VGAE to capture the molecular embedding and reconstruct a series of gene–miRNA association matrices, which contain rich genetic information and complex nonlinear relationships between molecules.

To simplify the model, we treat gene and miRNA nodes equally and construct the adjacency matrix ${\mathbf {A}} \in {\mathbb {R}^{(g+m) \times (g+m)}}$ for them based on gene–miRNA interaction matrix **W**_12_ as follows:


(1) \begin{eqnarray*} {{\bf A}} = \left[ {\begin{array}{*{20}{c}}{{{\bf 0}}}&{{{{\bf W}}_{12}}}\\ {{{{\bf W}}_{12}^{T}}}&{{{{\bf 0}}}} \end{array}} \right] \end{eqnarray*}

To represent dimensions uniformly, we reduce the dimension of the *i*-th type of attributes of genes **X**_1*i*_(*i* ∈ [1, *p*]) by Principal Component Analysis (PCA) (28), and then construct a molecular attribute data matrix ${{{\bf H}}_{i}}\in {\mathbb {R}^{(g+m) \times d_{ini}}}$ as follows:


(2) \begin{eqnarray*} {{{\bf H}}_{i}} = \left[ {\begin{array}{*{20}{c}}\mathop {\rm PCA}\nolimits ({{{\bf X}}_{1i}})\\ {{{{\bf 1}}}}\times {\mathop {\rm Median}\nolimits }({\mathop {\rm PCA}\nolimits }({{{\bf X}}_{1i}})) \end{array}} \right] \end{eqnarray*}

where *d*_*ini*_ is the dimension after PCA, ${{{\bf 1}}\in {\mathbb {R}^{m \times d_{ini}}}}$ is a matrix where all values are 1 and ${\mathop {\rm Median}\nolimits }(\cdot )$ is the median of matrix. ${\mathop {\rm Median}\nolimits }({\mathop {\rm PCA}\nolimits }({{{\bf X}}_{1i}}))$ aligns with the same dimension as genes and enables faster model convergence.

As for the *j*-th type of attributes of miRNAs **X**_2*j*_(*j* ∈ [1, *q*]), we can obtain the attribute ${{{\bf H}}_{i}}\in {\mathbb {R}^{(g+m) \times d_{ini}}}$ (*i* = *p* + *j*, *p* is the number of gene attribute types) as follows:


(3) \begin{eqnarray*} {{{\bf H}}_{i}} = \left[ {\begin{array}{*{20}{c}}{{{\bf 1}}}\times {\mathop {\rm Median}\nolimits }({\mathop {\rm PCA}\nolimits }({{{\bf X}}_{2j}})) \\ {\mathop {\rm PCA}\nolimits }({{{\bf X}}_{2j}}) \end{array}} \right] \end{eqnarray*}

With the association matrix **A** and multiple molecular attribute data matrices $\lbrace \mathbf {H}_{i}\rbrace _{i=1}^{v} (v=p+q)$ as input, HetFCM applies multiple VGAE to obtain the molecular embedding $\lbrace \mathbf {Z}_{i}\rbrace _{i=1}^{v}$ and reconstruct adjacency matrices $\lbrace \mathbf {A}^{^{\prime }}_{i}\rbrace _{i=1}^{v}$ (26):


(4) \begin{eqnarray*} \begin{aligned} \lbrace {{\bf Z}}_{i}, {\mathbf {A}^{^{\prime }}_{i}}\rbrace _{i=1}^{v} = {\mathop {\rm VGAE}\nolimits }({{{\bf A}}}, \lbrace \mathbf {H}_{i}\rbrace _{i=1}^{v}) \end{aligned} \end{eqnarray*}

The details of VGAE are given in Section 2 of the Supplementary Materials.

In this way, HetFCM obtains a series of association matrices $\lbrace \mathbf {A}^{^{\prime }}_{i}\rbrace _{i=1}^{v}$ by fusing different attribute data of genes and miRNAs $\lbrace \mathbf {H}_{i}\rbrace _{i=1}^{v}$, which have different biological significance from different perspectives. Finally, we extract the gene–miRNA association matrices $\lbrace \mathbf {W}^{^{\prime }}_{i}\rbrace _{i=1}^{v} \in \mathbb {R}^{g \times m}$ from the reconstructed ones $\lbrace \mathbf {A}^{^{\prime }}_{i}\rbrace _{i=1}^{v} \in \mathbb {R}^{(g+m) \times (g+m)}$ as follows:


(5) \begin{eqnarray*} \mathbf {W}^{^{\prime }}_{i} = \mathbf {A}^{^{\prime }}_{i}[1:g, g+1:g+m] \end{eqnarray*}

To reduce the noise of gene–miRNA association matrices $\lbrace \mathbf {W}^{^{\prime }}_{i}\rbrace _{i=1}^{v}$, we retain the top *t* gene–miRNA pairs among all unknown associations and use $\lbrace {\mathbf {W}}_{i}\rbrace _{i=1}^{v} \in \mathbb {R}^{g \times m}$ to denote the refined gene–miRNA association matrices. Notably, HetFCM can effectively fuse the new attribute data of genes or miRNAs by renewing $\lbrace \mathbf {W}_{i}\rbrace _{i=1}^{v}$, which gives rise to its good expandability.

### Step 2: adaptive weighted co-clustering

Co-clustering is a clustering technique that simultaneously performs clustering operations on both rows and columns of a data matrix and groups similar rows and columns together (8,29). The gene–miRNA association matrices generated by VGAE have two dimensions (genes and miRNAs), which make co-clustering well-suited to detect gene–miRNA co-modules. In Step 1, HetFCM generates multiple gene–miRNA association matrices, which embody different genetic information and have different relevance on co-module detection (30,31). To effectively integrate these association matrices and detect co-modules, we adopt an adaptive weighted co-clustering strategy to adeptly assign different weights to them as follows:


(6) \begin{eqnarray*} \begin{aligned} \begin{array}{c}\mathcal {O}_{1} = \sum \limits _{i = 1}^v {{{{\bf w}}_i}\left\Vert {{{{\bf W}}_i} - {{{\bf G}}_1}{{{\bf S}}_i}{{\bf G}}_2^T} \right\Vert _F^2} + \gamma \left\Vert {{\bf w}} \right\Vert _F^2 \\ {\rm {s.t. }}{{{\bf w}}^T}{\mathbf {1}} = 1,{w_i} \geqslant 0 \end{array} \end{aligned} \end{eqnarray*}

where ${\mathbf {G}_{1}} \in {\mathbb {R}^{g \times k}}$ and ${\mathbf {G}_{2}} \in {\mathbb {R}^{m \times k}}$ are the distributions of gene clusters and miRNA clusters, *k* is the number of co-clusters, and **S**_*i*_ is the harmonic matrix that implicitly constructs the mapping between gene clusters and miRNA clusters under the *i*-th gene–miRNA association matrix **W**_*i*_. **1** is a column vector with all values of 1 and **w** is a column vector where *w*_*i*_ is the adaptively assigned weight of the *i*-th association matrix. In this way, different genetic information in **W**_*i*_ is differently merged into the distributions of clusters **G**_1_ and **G**_2_ to guide the co-module detection. $\left\Vert {{\bf w}} \right\Vert _F^2$ is a regularization term to avoid the tendency to refactor matrices with small losses (32) and γ is the regularized coefficient.

Molecular expression data can be used to mine co-expression patterns, which guide the analysis of molecular function, regulation, and biological processes related to phenotypes (12,33,34). To gain the co-expression patterns, both gene expression data **X**_11_ and miRNA expression data **X**_21_ are factorized into a common basis matrix **B** and the distributions of gene/miRNA clusters **G**_*i*_(*i* = 1, 2) as follows:


(7) \begin{eqnarray*} \begin{aligned} \begin{array}{c}\mathcal {O}_{2} = \sum \limits _{i = 1,2} {\left\Vert {{{{\bf X}}_{i1}} - {{{\bf G}}_i}{{\bf B}}} \right\Vert _F^2} + \beta \sum \limits _{i = 1,2} {\left\Vert {{{{\bf G}}_i^T}{{{\bf G}}_{i}} - {{{\bf I}}}} \right\Vert _F^2} \\ {\rm {s.t.}}{{{\bf G}}_i} \ge 0,{{\bf B}} \ge 0 \end{array} \end{aligned} \end{eqnarray*}

where ${\mathbf {B}} \in {\mathbb {R}^{k \times n}}$ is the basis matrix. The second term is the orthogonalization constraints on **G**_*i*_ to reduce overlap between modules, where **I** is the identity matrix of *k*-dimension and β is the coefficient of orthogonalization constraints.

Since **G**_*i*_ is shared in Eqs. (6) and (7), we can project association data and molecular expression data toward **G**_*i*_ and effectively fuse them. Finally, the objective function can be expressed as:


(8) \begin{eqnarray*} \begin{aligned} {\mathcal {O}} &= {\mathcal {O}_{1}} + \alpha {\mathcal {O}_{2}} \hfill \\ &= \sum \limits _{i = 1}^v {{{{\bf w}}_i}\left\Vert {{{{\bf W}}_i} - {{{\bf G}}_1}{{{\bf S}}_i}{{\bf G}}_2^T} \right\Vert _F^2} + \alpha \sum \limits _{i = 1,2} {\left\Vert {{{{\bf X}}_{i1}} - {{{\bf G}}_i}{{\bf B}}} \right\Vert _F^2} \\ & + \beta \sum \limits _{i = 1,2} {\left\Vert {{{{\bf G}}_i^T}{{{\bf G}}_{i}} - {{{\bf I}}}} \right\Vert _F^2} + \gamma \left\Vert {{\bf w}} \right\Vert _F^2 \\ &{\text{s.t. }}{\mathbf {G}_{i}} \geqslant 0,{\mathbf {S}_{i}} \geqslant 0, {{{\bf w}}^T}{\mathbf {1}} = 1,{{{\bf w}}_i} \geqslant 0 \hfill \\ \end{aligned} \end{eqnarray*}

where α is the weight that controls the influence of molecular expression data. In this way, HetFCM differently integrates the gene–miRNA association matrices and molecular expression data, enhances the biological information of cluster distribution matrix **G**_*i*_ and further boosts the co-module detection. Since the optimization of Eq. (8) is not convex, we adopt the iterative updating strategy by alternatively optimizing one variable while fixing others to approximately solve this problem, and the optimization details are reported in Section 3 of the Supplementary Materials.

### Step 3: co-module discovery

In co-module detection, some molecules may participate in multiple functional modules simultaneously, while other molecules do not participate in any module. Thus, to avoid the bias caused by matrix factorization at the cluster level and accurately identify the functional co-modules, a threshold-based strategy (23) is applied to detect co-modules based on the cluster distribution matrices **G**_1_ and **G**_2_ obtained from Step 2. The *z*-score value of each column element of **G**_1_ and **G**_2_ is as follows:


(9) \begin{eqnarray*} {z_{ij}} = \frac{{{g_{ij}} - {\mu _j}}}{{{\sigma _j}}} \end{eqnarray*}

where *g*_*ij*_ is [**G**_1_]_*ij*_ (or [**G**_2_]_*ij*_), μ_*j*_ is the average value of the *j*-th column in **G**_1_ (or **G**_2_) and σ_*j*_ is the standard deviation. If *z*_*ij*_ exceeds a given threshold *T*_1_ (or *T*_2_), gene *i* (or miRNA *i*) is assigned to co-module *j*, then all gene–miRNA functional co-modules can be detected. We use cluster indicator matrix ${\mathbf {F}_{1}} \in {\mathbb {R}^{g \times k}}$ and ${\mathbf {F}_{2}} \in {\mathbb {R}^{m \times k}}$ to represent the module indicator of genes and miRNAs, respectively. If gene *i* is assigned to co-module *j*, then **F**_1_(*i*, *j*) = 1, otherwise, **F**_1_(*i*, *j*) = 0, so as for miRNAs. We want to remark that HetFCM can be extended to detect multi-layer functional modules, such as gene–miRNA–lncRNA functional modules demonstrated in Figure 5. The procedure of HetFCM is described in the Algorithm table and the time complexity is discussed in Section 4 of the Supplementary Materials.


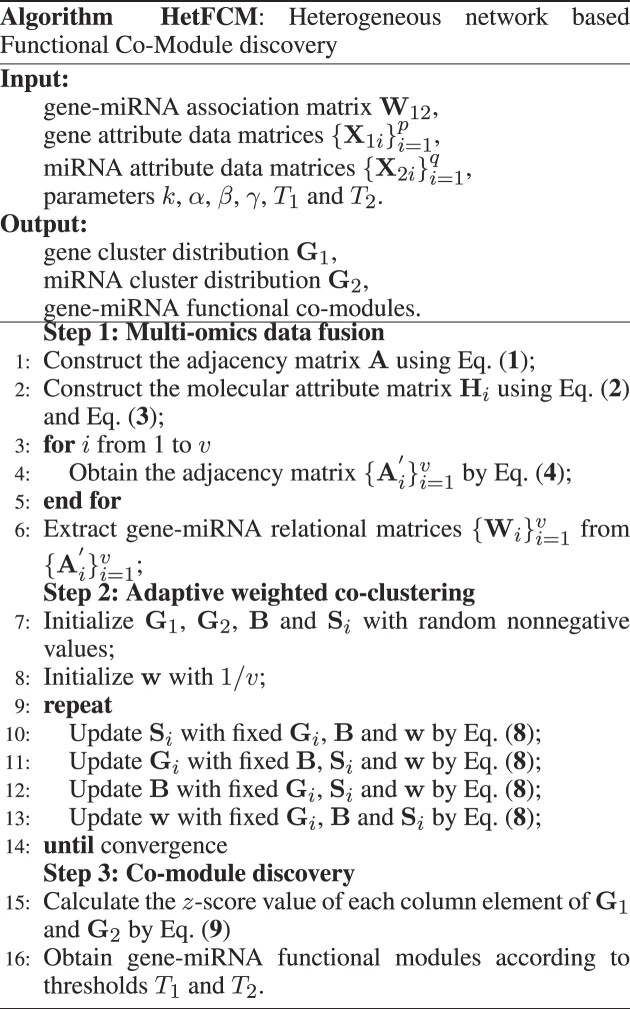


## Results

### Compared methods and parameter settings

To study the co-module detection performance of HetFCM, we compare it against two types of mainstream methods: enumeration-based and collaborative clustering-based methods. Pseudo3D (20) is a representative enumeration-based method that detects gene–miRNA modules, while the other five methods are based on collaborative clustering. SNPLS (35) incorporates a sparse gene–gene interaction network to the partial least square model to identify joint gene–drug patterns. TsRFR (22) obtains the regulatory matrix between miRNAs and mRNAs based on regularized factor regression and extracts miRNA–mRNA co-modules by factorizing the regulatory matrix into two low-rank matrices. HOGMHC (36) is a high-order graph matching model with hypergraph constraints to detect gene-drug modules. NetNMF (23) is a non-negative matrix factorization framework that detects gene–miRNA co-modules based on co-expression networks of heterogeneous molecules. JONMF (37) employs multiple non-negative matrix factorization with orthogonal constraints to detect lncRNA–mRNA co-modules using single omics data. All these methods can be adapted to detect gene–miRNA co-modules. For compared methods that need to specify the number of clusters, we uniformly fix the number of clusters as 30 for performance comparison. This number is determined by *k*-means on datasets and suitable for these methods also. The other parameters are the default values in the public code or optimized within the range suggested by the respective literature. The detailed parameter analysis and settings of HetFCM are given in Supplementary Figure S1–S6 in Section 6 of the Supplementary Materials. In particular, the adaptively assigned weight **w** for association matrices are also analyzed in the analysis of parameter γ in Section 6 of the Supplementary Materials, which reflects the importance of different attribute data.

### Performance evaluation

We comprehensively evaluate the performance of HetFCM and compared methods at both molecule and module levels.

For the molecule level evaluation, we measure the capability of all methods on identifying genes and miRNAs associated with a particular phenotype/function. We repeat all methods on Human dataset 10 times and report the average and variance of AUROC (Area Under the Receiver Operating characteristic Curve), AUPRC (Area Under the Precision-Recall Curve) and *F*1-score in Table 1. HetFCM achieves the best performance at both the gene and miRNA levels, while JONMF and HOGMHC hold the runner-up performance. That is because these three methods fuse more network data than other methods. HetFCM outperforms JONMF and HOGMHC, which confirms that multi-omics attributes of molecules can guide the detection of key molecules. SNPLS does not fuse gene–miRNA association data, which is not conducive to the detection of cancer-related molecules, so it loses to others. The results obtained by NetNMF and TsRFR are not outstanding. That is because they only use molecular expression data. The above results indicate that the fusion of multi-omics data completes the cross-layer association matrices and contributes to mining key molecules associated with human breast cancer.

**Table 1.** The molecular level evaluation results of HetFCM with compared methods on Human dataset

Levels	Methods	AUROC	AUPRC	*F*1-score
gene	TsRFR	0.7446 ± 0.0046	0.3992 ± 0.0066	0.2686 ± 0.0067
	SNPLS	0.6814 ± 0.0158	0.3589 ± 0.0185	0.2090 ± 0.0196
	NetNMF	0.5167 ± 0.0118	0.1876 ± 0.0068	0.1871 ± 0.0066
	HOGMHC	0.7412 ± 0.0079	0.3756 ± 0.0083	0.2129 ± 0.0153
	JONMF	0.6864 ± 0.0110	0.3865 ± 0.0162	0.2259 ± 0.0097
	HetFCM	**0.7775 ± 0.0018**	**0.4500 ± 0.0056**	**0.2351 ± 0.0045**
miRNA	TsRFR	0.4902 ± 0.0689	0.0926 ± 0.0146	0.1513 ± 0.0064
	SNPLS	0.6817 ± 0.0573	0.2535 ± 0.0534	0.1938 ± 0.0112
	NetNMF	0.6957 ± 0.0092	0.1732 ± 0.0052	0.1645 ± 0.0034
	HOGMHC	0.7849 ± 0.0132	0.3104 ± 0.0178	0.1801 ± 0.0035
	JONMF	0.7995 ± 0.0049	0.3385 ± 0.0156	0.1950 ± 0.0117
	HetFCM	**0.8446 ± 0.0039**	**0.4803 ± 0.0067**	**0.2594 ± 0.0082**

**Figure 2.**
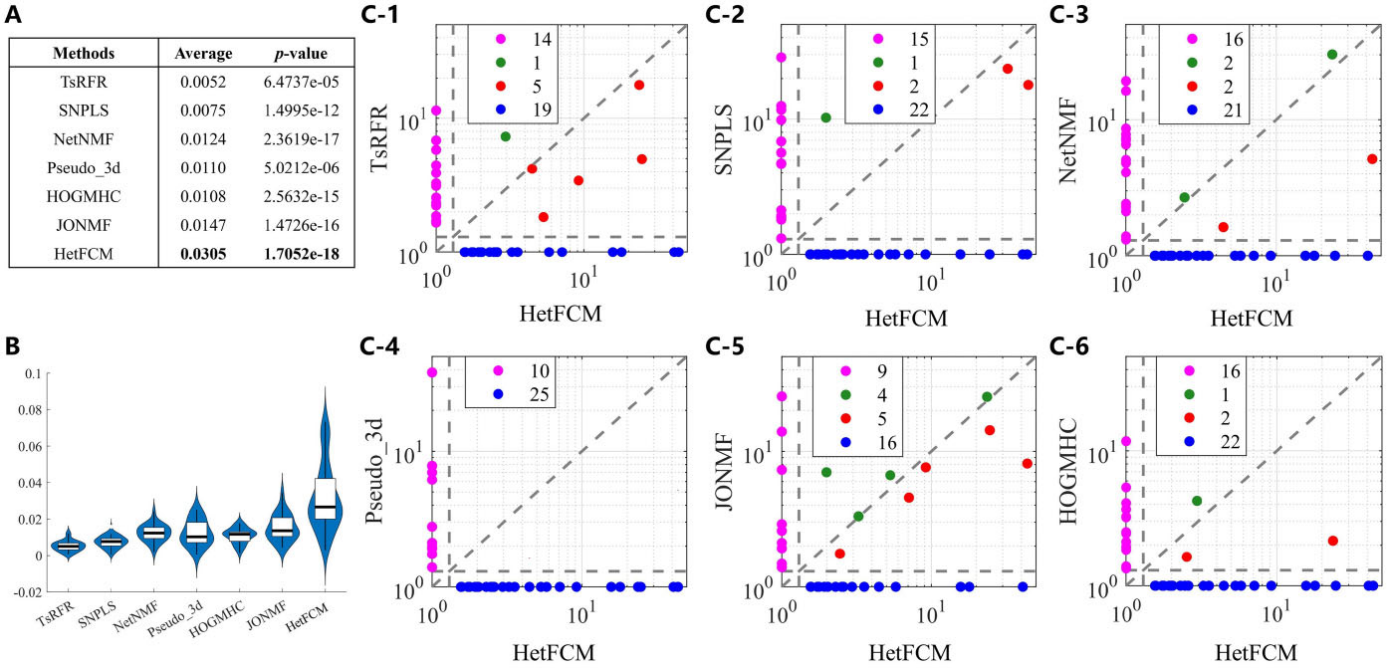
Performance comparison of HetFCM and other methods at module level evaluation on Human dataset. (**A**) reports the modularity of gene–miRNA co-modules detected by HetFCM and other methods. (**B**) shows the distribution of modularity of co-modules detected by all methods. (**C**) reveals the enrichment degree of biological processes. The horizontal and vertical axis represents the evaluation scores of HetFCM and other methods, respectively. The horizontal and vertical dash lines on the plot both represent the enrichment threshold (Benjamini Hochberg-corrected *p*-value=0.05). Magenta points denote enriched biological processes detected only by the compared method while blue ones are only by HetFCM. Both the green and red points represent biological processes simultaneously enriched by HetFCM and compared methods. Red points under the diagonal dash line mean that HetFCM has higher evaluation scores than compared methods, while green points above the diagonal dash line mean the opposite.

For module level evaluation, we adopt modularity (defined in Section 5 of the Supplementary Materials) and the enrichment degree of biological processes to evaluate the gene–miRNA co-modules from the perspective of network topology and functional significance. Figure 2A gives the average value of modularity and the statistical significance *p*-value (defined in Section 5 of the Supplementary Materials) on Human dataset, while Figure 2B shows the distribution of modularity for all co-modules. From Figure 2A and B, the modularity of co-modules detected by HetFCM is more significant than those of compared methods and the modularity statistical significance is also extremely better, which suggests that HetFCM can utilize multi-omics data to complete the gene–miRNA association network and acquire gene–miRNA co-modules with more dense topology.

**ClusterProfiler** package (38) in **R** is used to test the Gene Ontology (GO) biological processes of each module at the gene level for functional significance. We filter out enriched biological processes whose *p*-value >0.05, calculate the –log_10_ (Benjamini Hochberg-corrected *p*-value) of each enriched biological process as evaluation score, and choose the highest value among all modules as its final evaluation score for each method. As shown in Figure 2C, the number of blue points is larger than that of magenta ones, indicating that HetFCM can detect more modules than the compared methods. The number of red points is bigger than that of green ones, indicating HetFCM detects more BP terms with better enrichment degrees than rivals. HetFCM detects more modules with an evaluation value ≥ 10 than all compared methods. In conclusion, the results show that the enriched biological processes of HetFCM are more significant than those of compared methods. The superiority of HetFCM and more analyses are discussed in Section 7 of Supplementary Materials.

Experimental results on Maize dataset are shown in Supplementary Figure S7 in Section 8 of the Supplementary Materials. Compared to the results on Human dataset, we can see that the modularity of detected co-modules on Maize dataset is clearly smaller, this is because the gene–miRNA interaction network of Maize dataset is much sparser than that of Human dataset. Other results give similar conclusions to those on Human dataset.

We also analyze the importance of each omic data and set up four variants for HetFCM, which separately disregard sequence data, gene-gene interactions, molecular expression data and multiple gene–miRNA association matrices. The results are shown in Supplementary Figures S8 and S9 of the Supplementary Materials, which prove that both multi-omics attribute data and gene–miRNA associations are helpful for module detection and can make the function of detected modules more significant. In addition, HetFCM can flexibly fuse other omics data, which validates its expandability. These results and analysis are discussed in Section 9 of the Supplementary Materials.

### Discoveries on human breast cancer dataset

On the human breast cancer dataset, 30 gene–miRNA co-modules detected by HetFCM covers an average of 47 genes and 9 miRNAs (Supplementary Table S1). In this section, we analyze these gene–miRNA co-modules from different perspectives.

**Figure 3.**
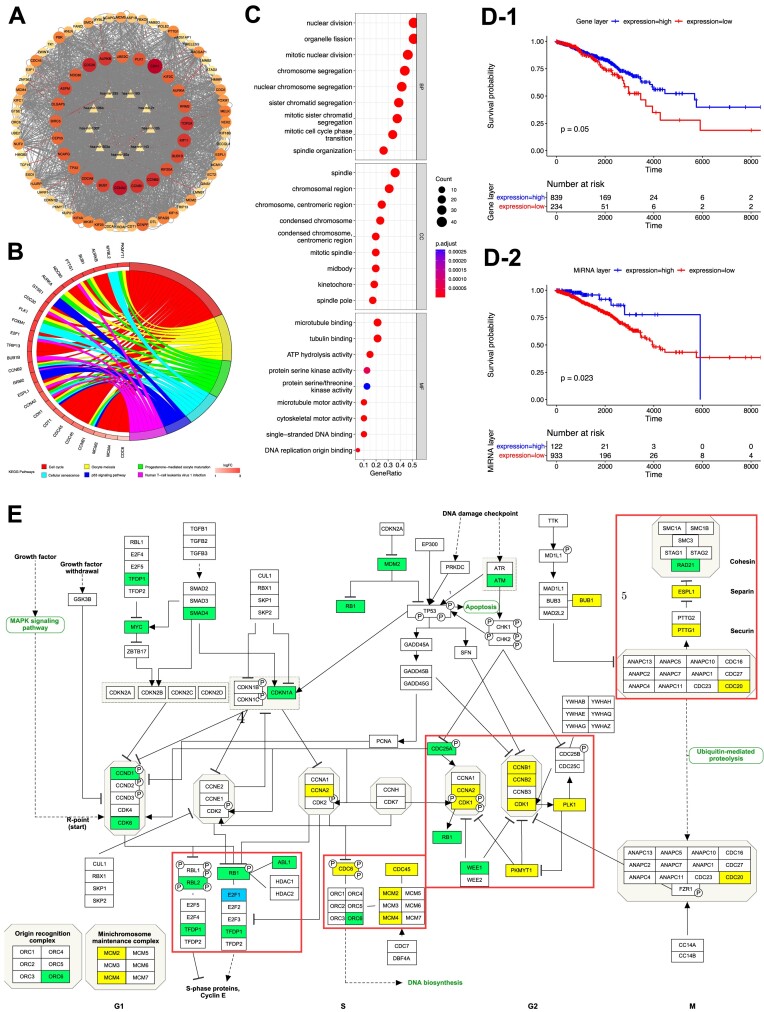
Information of gene–miRNA co-module 5 on Human breast cancer dataset. (**A**) shows interactions between molecules in co-module 5. (**B**, **C**) separately shows the result of enrichment analysis of KEGG pathway/GO on the gene layer. (**D**) reports Kaplan–Meier survival analysis results on the gene layer and miRNA layer. (**E**) shows the WikiPathway (https://www.wikipathways.org/) WP179 (cell cycle) enriched by gene–miRNA co-module 5. The rectangle boxes are the genes involved in the pathway, while the yellow boxes are the genes in the gene layer, the green boxes are the genes corresponding to the miRNA layer, and the blue boxes are overlapped genes of the gene and miRNA layers. The red rectangle boxes indicate the regulatory patterns of genes and miRNAs target genes.

**Figure 4.**
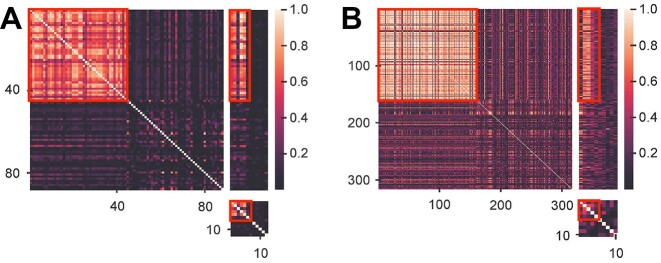
Heat maps of gene–miRNA co-modules (red rectangle boxes). We extend the heat map by randomly selecting the same number of genes and miRNAs for comparison. (**A**) The heat map of the gene–miRNA co-module 20 on Human breast cancer dataset. (**B**) The heat map of the gene–miRNA co-module 21 on Maize dataset.

**Figure 5.**
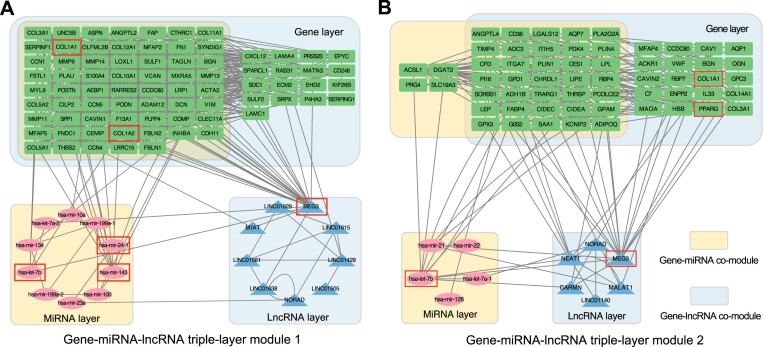
Gene-miRNA–lncRNA triple-layer modules detected by HetFCM. The yellow regions represent the gene–miRNA co-module, the blue regions represent the gene-lncRNA co-module, and the overlapped area corresponds to genes contained in both co-modules. The association data sources are listed in Supplementary Table S2 in Section 12 of the Supplementary Materials. (**A**) Gene–miRNA–lncRNA triple-layer module 1, composed of gene–miRNA co-module 11 and gene-lncRNA co-module 7. (**B**) Gene–miRNA–lncRNA triple-layer module 2, composed of gene–miRNA co-module 14 and gene-lncRNA co-module 21.

**Figure 6.**
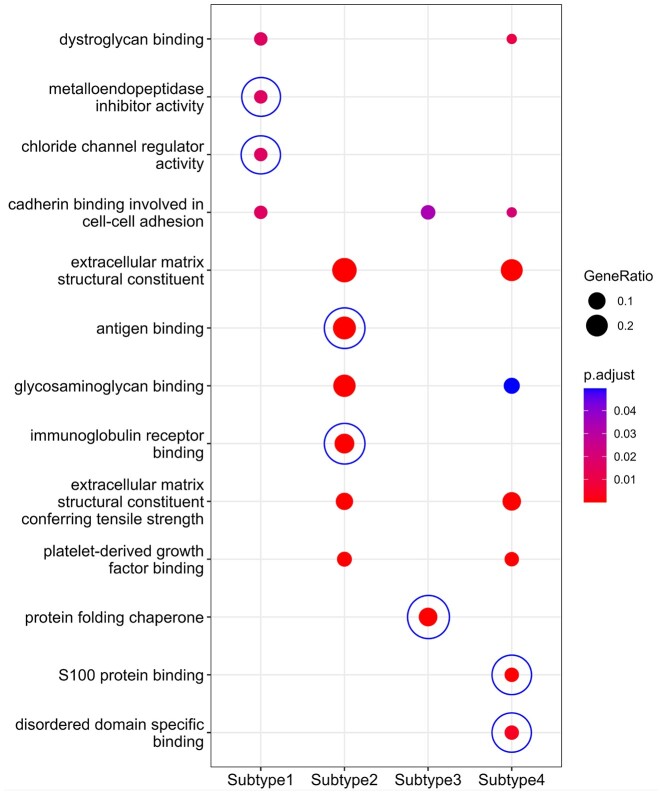
Enrichment analysis of genes in co-modules associated with four specific breast cancer subtypes. Bubbles with blue circles indicate subtype-specific functions.

- *HetFCM can detect co-modules with dense topology and phenotypic associations*.
  As shown in Figures 2A and 3A, the co-modules detected by HetFCM have high modularity values and molecules in co-modules have dense topology, which reflect the genes and miRNAs in co-modules synergistically interact to perform biological functions. In addition, we construct co-expression networks using Pearson correlation coefficient based on gene expression data as well as miRNA expression data, and analyze the co-expression patterns of one detected co-module (co-module 20). Figure 4A is the heat map of co-module 20 with 44 genes and 6 miRNAs and these molecules in the co-module have distinct blocks compared to randomly selected molecules, suggesting the co-modules detected by HetFCM have significant co-expression patterns. The molecules in the co-modules detected by HetFCM are not only related in topology but also closely associated with the phenotype.
  We list the average precision of genes and miRNAs associated with breast cancer in the co-modules detected by each method. As shown in Table 2, the co-modules detected by HetFCM have the largest precision. Compared with the best rival JONMF, HetFCM improves the precision by 9.4% and 8.6% at gene and miRNA levels, respectively, suggesting a good modular mining capability. In conclusion, HetFCM can detect co-modules with strong modularity and high phenotypic associations. The molecules in the co-modules have compact relations and may synergistically regulate the phenotypes.
- *HetFCM can detect co-modules with significant functions in gene and miRNA layers*.
  To study the functions of co-modules detected by HetFCM, we perform Biological Processes (BP) term enrichment analysis and pathway enrichment analysis at the gene level and miRNA level. In total, 28 gene layer modules (93%) and 30 miRNA layer modules (100%) are enriched in at least one BP term or pathway (Supplementary Table S2 and S3). We discuss some significant co-modules below.
  For gene layer modules, the detected gene–miRNA co-module 5 contains 82 genes and 12 miRNAs, enriched to 271 BP terms with *p*-value<0.05. Among all genes, 73 genes (89%) are associated with breast cancer according to DisGeNET database (http://www.disgenet.org/) (39). Figure 3 B gives the result of enrichment analysis of KEGG pathway on the gene layer, including *P53 signaling pathway*, which represents one of the most important and extensively studied tumor suppressors and relates to breast cancer (40,41). The most significant enriched BP term is GO:0140014 (mitotic nuclear division) in co-module 5. Mitotic nuclear division, organelle fission and mitotic cell cycle phase transition are closely related processes, all of which occur in cell division and proliferation. During these processes, some alterations and modifications appear, such as genetic variations and epigenetic events, which may cause cancer cell initiation and progression (35,42). GO:0048285 (organelle fission) and GO:0044772 (mitotic cell cycle phase transition) are also enriched in co-module 5 as shown in Figure 3C. In addition, GO:0051304 (chromosome segregation) is significantly enriched in this co-module, which is an important process that is related to tumorigenesis (17). Tanak *et al.* reported that the loss or gain of chromosomes, known as chromosome instability, is associated with many cancer cells and might arise from a lesion in the chromosome segregation machinery (43). In summary, the gene layer of co-module 5 is closely related to cancer-related cellular processes, indicating HetFCM can effectively detect gene modules with significant functions.
  For gene layer modules, the most significant enriched Wikipathway (an open science platform for biological pathways contributed, updated and used by the community) is WP2446 (retinoblastoma gene in cancer) in co-module 2. Retinoblastoma protein is a product of the retinoblastoma tumor suppressor gene, whose expression is highly prevalent in luminal breast cancers (44). As shown in Supplementary Figure S10 in Supplementary Materials, the rectangle boxes represent the genes involved in this pathway, while the yellow boxes represent the genes that are in the gene layer of co-module 2. From this pathway, we can observe that co-module 2 can cover the key driver genes that regulate the development and progression of breast cancer, indicating that the gene modules detected by HetFCM are highly correlated with cancer pathways. WP179 (cell cycle) is also enriched in co-module 2. There are 13 genes enriched in WP179 (cell cycle) while 15 genes enriched in WP2446 (retinoblastoma gene in cancer) (as shown in Supplementary Table S3). Among these genes, 10 genes are the same, indicating that WP179 (cell cycle) and WP2446 (retinoblastoma gene in cancer) may cross-talk and jointly trigger cancers. These results demonstrate that the co-modules detected by HetFCM can guide the discovery of potential cooperative driver pathways.
  For miRNA layer modules, we analyze the function of target genes of all miRNAs. In order to more accurately determine the target genes of these miRNAs, we only reserve the target genes whose number of references ≥2 (https://mirtarbase.cuhk.edu.cn) (45). The most significantly enriched BP term of miRNA layer modules is GO:0044843 (cell cycle G1/S phase transition) in gene–miRNA co-module 30. The accurate transition of the cell cycle from G1 phase to S phase is crucial for the control of eukaryotic cell proliferation, and its misregulation promotes the development of tumors (46). The most significantly enriched Wikipathways are WP1984 (integrated breast cancer pathway) in co-module 20 and WP2261 (glioblastoma signaling pathways) in co-module 14. WP1984 (integrated breast cancer pathway) no longer exists in the latest version of Wikipathways, so we show WP2261 (glioblastoma signaling pathways) in Supplementary Figure S11 of Supplementary Materials. We can see that a number of target genes are enriched in glioblastoma signaling pathways. The dysregulation of glioblastoma signaling pathways can lead to glioblastoma, which has a very complex pathogenesis that involves mutations and alterations of several key cellular pathways, such as cell proliferation, survival, migration, apoptosis and angiogenesis (47). These results also confirm that the miRNA modules detected by HetFCM are indeed associated with breast cancer.
- *Genes and miRNAs in co-module cooperatively contribute to biological functions or phenotypes*
  In order to verify the cooperative relations between the gene layer and miRNA layer in co-modules, we analyze the BP terms and pathways co-enriched by gene layer and miRNA layer. Among 21 of 30 gene–miRNA co-modules, the gene layer modules and miRNA layer modules are co-enriched into at least one common BP term or pathway, and they are strongly associated with breast cancer.
  For BP terms, gene and miRNA modules in co-module 10 share GO:0050678 (regulation of epithelial cell proliferation). Epithelial cell proliferation is a risk factor for cancer (48), and its regulation disorder is more likely to lead to cancer. Gene and miRNA modules in co-module 23 are co-enriched into GO:0022612 (gland morphogenesis). Existing studies suggest that a better understanding of genetic pathways involved in mammary gland morphogenesis has profound implications for breast cancer (49,50).
  For pathways, gene and miRNA modules in co-module 5 share WP179 (cell cycle) as shown in Figure 3E. The molecules in co-module 5 cover most molecules in cell cycle pathway (labeled yellow, green and blue boxes). The regulation patterns of genes and miRNA target genes in co-module 5 are highlighted in the red rectangle box in Figure 3E, which synergistically affect cell cycle. The deregulation of cell cycle is a common feature of human cancer (51). Breast cancer cells often exhibit dysregulation in the cell cycle, leading to uncontrolled cell growth and tumor formation (52–54). This finding suggests that co-module 5 is closely associated with breast cancer. In addition, both gene and miRNA layer modules in co-module 20 are enriched into WP3929 (chemokine signaling pathway), as shown in Supplementary figure S12 of Supplementary Materials. The development of primary tumours and metastases are influenced by chemokine signaling pathway and their receptors. Many cancers have a complex chemokine pathway that impacts tumor growth, angiogenesis, invasion, and metastasis (55). In breast cancer pathogenesis, chemokines and their receptors are involved in the metastatic spread of cancer cells and tumor-stroma interactions (56). These results indicate that the genes and miRNAs in co-modules detected by HetFCM can collaboratively contribute to biological functions or phenotypes, which provides a holistic insight of regulations among pathogenic molecules.
  To further evaluate the correlation between genes and miRNAs and their effect on breast cancer, we perform survival analysis based on real case data. For this purpose, we download the clinical data from TCGA, filter out samples without survival time or not recorded in the expression data, and retain 1,055 samples for the Kaplan–Meier survival analysis. We divide these samples into two groups based on the lower and higher molecular expression levels than the cutpoint, which is obtained by $surv\_cutpoint$ function in **R** package **survminer** (57). We then perform the Kaplan–Meier survival analysis using **R** package **survival** (58) to compare the survival characteristics of the two groups with significance determined by the log-rank test. As shown in Figure 3D and S13B in Supplementary Materials, notably, the curves demonstrate that the higher expression level of these two co-modules both at gene layer and miRNA layer are significantly related with decreased survival rates of breast cancer patients, indicating that these co-modules play an important role in the pathogenesis of breast cancer. These results manifest that the functional co-modules detected by HetFCM are of great significance for the analysis of the etiology of clinical cases. The gene–miRNA co-modules detected by HetFCM also can be verified by existing literature, such as co-module 17 as shown in Supplementary Figure S13 of Supplementary Materials, and these co-modules are discussed in Section 11 of Supplementary Materials.
- *HetFCM can be extended to detect multi-layer functional modules*.
  The multi-layer molecular functional modules can reveal the complex regulatory network of different types of molecules, and the intro/inter interactions between these molecules synergistically affect the function of cells and the development of diseases. Studying multi-layer molecular functional modules can decipher the complex biological mechanisms and plays a pivotal role in the comprehensive analysis of phenotype-related mechanisms.
  The gene–miRNA–lncRNA triple-layer module represents a typical multi-layer molecular functional module. To verify the extensibility of HetFCM for the identification of multi-layer functional modules, we employ gene–miRNA–lncRNA triple-layer module detection as an illustrative example. First, we obtain and process lncRNA-related data, with details provided in Section 12 of the Supplementary Materials. Subsequently, HetFCM is executed to obtain the gene-lncRNA co-modules. We calculate the Jaccard Similarity between gene layer modules in gene–miRNA co-modules and gene-lncRNA co-modules and select the two co-module pairs with the highest similarity. Finally, we interconnect the gene–miRNA co-module and gene-lncRNA co-module to form a gene–miRNA–lncRNA triple-layer module, as illustrated in Figure 5.
  The molecular precision associated with breast cancer in three-layer functional modules 1 and 2 are 0.6195 and 0.7079, respectively. In the two modules, the key molecules with the largest degree, betweenness and closeness are shown in Table 3. COL1A1 and COL1A2 are associated with breast cancer. The research in mouse models of breast cancer indicates that COL1A1 is a key extracellular matrix gene and is identified with copy number alterations through integrative in vitro and in vivo studies (59), while COL1A2 methylation is associated with ER/PR receptor status in breast cancer cohort (60). PPARG is associated with breast cancer and serves as a prognostic factor for this disease (61). The dysregulation of hsa-let-7b is associated with breast cancer (62) and MEG3 acts as a tumor suppressor in breast cancer development and progression (63). Other molecules can also be validated for their associations with breast cancer through relevant literature. In gene–miRNA–lncRNA functional module 1, lncRNA MEG3 contributes to the development of cardiac fibrosis while the expression of miRNA hsa-mir-100 is associated with fibroblasts in breast cancer (64). Gene MMP13 is a target gene of hsa-mir-100 (65) and can be upregulated by MEG3 (66). These three molecules with different types may synergistically trigger breast cancer. The above results suggest that HetFCM can be used to detect multi-layer functional modules related to breast cancer.
- *HetFCM can find the co-modules associated with cancer subtypes*.
  Cancer subtypes refer to histopathologically indistinguishable subgroupings within a particular cancer type, substantially impacted by different functional modules (70). Therefore, identifying functional co-modules related to specific cancer subtypes can reveal the heterogeneity of subtypes and facilitate the development of subgroup-specific therapies (71).
  To demonstrate the capability of HetFCM for detecting co-modules associated with cancer subtypes, we take breast cancer subtype identification as an example. Since there are four main subtypes in breast cancer (Luminal A, Luminal B, Basal and HER2) (72), we set the number of gene–miRNA co-clusters *k* = 4 (Supplementary Table S4). Table 4 lists the evaluation results of molecules in co-modules separately related to the four subtypes. The precision of genes and miRNAs is relatively high, indicating HetFCM can accurately detect molecules associated with breast cancer. However, the recall is relatively low, that is because the number of molecules in the co-module is quite small while the number of molecules associated with breast cancer is relatively large.
  To investigate the functional co-modules associated with breast cancer subtypes, we perform functional enrichment analysis based on the genes in the co-modules. As demonstrated in Figure 6, the subtype-specific genes are enriched in distinct GO terms. For subtype 1, the associated distinct GO terms include metalloendopeptidase inhibitor activity and chloride channel regulator activity. Metalloendopeptidases, such as matrix metalloproteinases (MMPs), play a critical role in the degradation of the extracellular matrix, which is essential for cancer cell invasion and metastasis (73). Chloride channels are involved in the regulation of cell volume, and their dysregulation can affect cancer cell proliferation, migration, and invasion (74). Subtype 2 has two distinct enriched GO terms, antigen binding and immunoglobulin receptor binding. These two functions can help the immune system recognize and attack cancer cells and provide a critical foundation for the development of various immunotherapy strategies (75,76). The specific GO term for subtype 3 is protein folding chaperone. Protein folding chaperone, i.e. the chaperonin-containing tailless complex polypeptide 1 (CCT), affects cell division, directed cell migration and invasion, fundamentally linked to cancer (77). For subtype 4, the associated distinct GO terms are S100 protein binding and disordered domain specific binding. S100 protein family modulates cellular responses by functioning as intracellular Ca2+ sensors and extracellular factors, which contribute to tumorigenic processes such as cell proliferation, metastasis, angiogenesis and immune evasion (78). Intrinsically disordered N-terminal transactivation domain can long-range regulate p53 DNA binding and play a crucial role in cell biology and cancer research (79). These co-modules associated with different cancer subtypes have distinct functions, which can provide new insights into the diagnosis and treatment of specific subtypes.

**Table 2.** The average precision of molecules associated with breast cancer in co-modules detected by HetFCM and compared methods

Levels	TsRFR	SNPLS	NetNMF	Pseudo_3d	JONMF	HOGMHC	HetFCM
gene	0.5186	0.3536	0.4289	0.2274	0.5829	0.4068	**0.6379**
miRNA	0.0673	0.2546	0.3407	0.2250	0.5967	0.3963	**0.6481**

**Table 3.** The key molecules with the largest degree, betweenness and closenes in two gene–miRNA–lncRNA functional module

Module	Index	Gene	miRNA	lncRNA
Gene-miRNA–lncRNA	Degree	COL1A1 (59,67)	hsa-mir-143 (68)	MEG3 (63)
Functional module 1	Betweenness	COL1A2 (60)	hsa-mir-24-1 (69)	MEG3 (63)
	Closeness	COL1A2 (60)	hsa-let-7b (62)	MEG3 (63)
Gene-miRNA–lncRNA	Degree	PPARG (61)	hsa-let-7b (62)	MEG3 (63)
Functional module 2	Betweenness	COL1A1 (59,67)	hsa-let-7b (62)	MEG3 (63)
	Clossness	PPARG (61)	hsa-let-7b (62)	MEG3 (63)

**Table 4.** The evaluation results of genes and miRNAs within four co-modules separately associated with four breast cancer subtypes

Levels	Methods	Co-module 1	Co-module 2	Co-module 3	Co-module 4
gene	precision	0.7692	0.6230	0.8485	0.9184
	recall	0.0145	0.0183	0.0135	0.0217
miRNA	precision	0.5714	0.7273	0.6667	0.7273
	recall	0.0667	0.0444	0.0222	0.0444

### Discoveries on Maize dataset

On Maize dataset, 30 detected gene–miRNA co-modules have an average of 75 genes and 10 miRNAs per module, 30 gene layer modules (100%) are enriched in at least one BP term (S5 and Supplementary Table S6).

**Figure 7.**
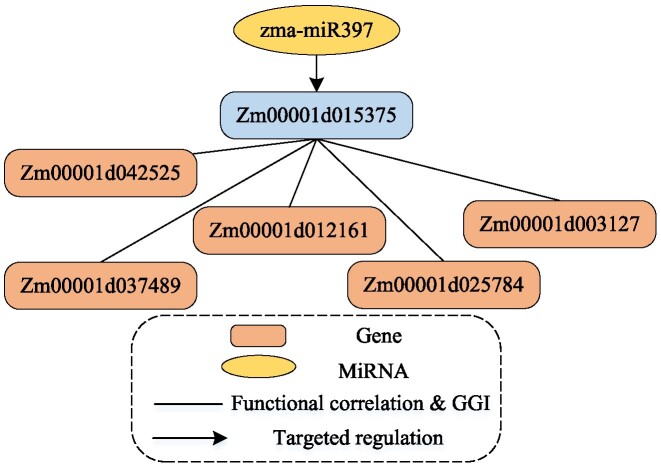
The potential target genes of zma-miR397 in co-module 26. The blue box is the known target gene, while the orange boxes are the potential targets, and these potential genes have strong interactions and high functional similarity with the known target.

**Figure 8.**
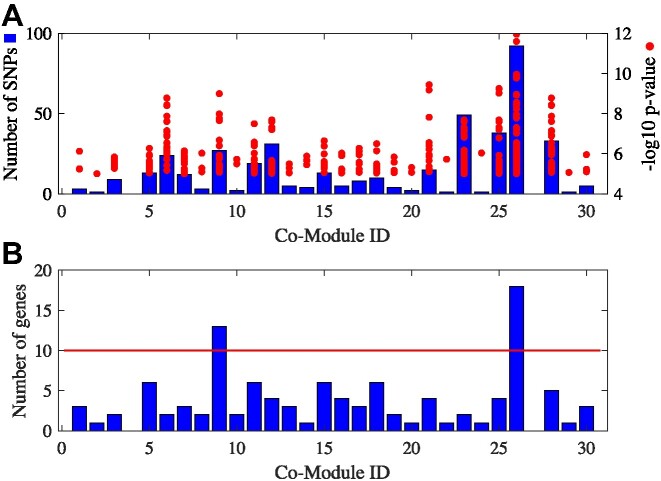
Statistics of SNPs related to oil content in each co-module on Maize dataset. (**A**) represents the number of SNPs and their statistical significance. (**B**) represents the number of genes these SNPs are located at.

- *HetFCM can detect co-modules with dense topology and significant functions*.
  The gene–miRNA co-modules detected by HetFCM on Maize dataset also have dense topology. As shown in Supplementary Figure S7A in Supplementary Materials, the co-modules detected by HetFCM have high modularity values. The heat map of co-module 21 in Figure 4B also demonstrates the co-modules detected by HetFCM have significant co-expression patterns. The molecules in the co-modules have strong correlations, and they are more likely to cooperatively affect the phenotype.
  For the functions of gene layer modules, the most enriched GO terms are GO:0022625 (cytosolic large ribosomal subunit), GO:0001510 (RNA methylation) and GO:0022627 (cytosolic small ribosomal subunit) in gene layer of co-module 26. Cytosolic ribosomal subunit is a macromolecular complex that contains both RNA and protein molecules, and it is related to protein synthesis (80). RNA methylation is an important post-transcriptional modification that influences gene regulation, which plays a critical role in plant development (81). In addition, we find a gene module that associates with the maize phenotype of interest, lipid storage, in co-module 16. The oleosins represent the lipid storage proteins that are part of the structural unit of the single layer membrane surrounding lipid bodies in seed and are thought to be important for oil body stabilization in the cytosol(82). In co-module 16, Zm00001d033612 (ole4) is an oleosin-like gene associated with organism development (83,84), highly up-regulated in maize endosperm periphery (85) and associated with seed oil storage (86). Zm00001d025833 (php20719a) encodes a caleosin family member and likely plays a role in seed oil body formation (87), which would be expected to directly affect embryo development more than endosperm development (88). In addition, Zm00001d027258 is linked to the generation of lipid bilayers (89). The above evidence shows that module 16 is closely related to lipid storage and suggests that HetFCM can detect new functional modules associated with key traits and assist crop breeding.
- *HetFCM can find out new regulations between genes and miRNAs*.
  HetFCM can uncover new regulations between genes and miRNAs in gene–miRNA co-modules. As an illustrative example, zma-miR397, an important miRNA of maize, relevant with drought tolerance and salt stress treatment (90,91), has been identified with its target genes as well as potential target genes in gene–miRNA co-module 26 by HetFCM.
  Zm00001d015375 (umc2298) is a known target gene of zma-miR397 (92) in co-module 26 and associated with drought tolerance (93). In co-module 26, there are 5 genes with strong interaction (*p*-value = 1.0979 × 10^−4^, Wilcoxon rank sum test) and high functional similarity (*p*-value = 1.7484 × 10^−3^, Wilcoxon rank sum test) with Zm00001d015375 as shown in Figure 7. Zm00001d042525 (rpl5a) and Zm00001d012161 (rpl5b) are associated with GO:0009628 (response to abiotic stimulus), GO:0006950 (response to stress), GO:0006970 (response to osmotic stress) and GO:0009651 (response to salt stress). When plants are exposed to drought conditions, the osmotic pressure and salt stress regulation mechanisms in the body will be activated. As such, it can be inferred that zma-miR397, Zm00001d015375, Zm00001d012161 and Zm00001d042525 may synergistically regulate the pathway related to drought tolerance. Notably, Yang *et al.* had found that miR397 plays an important role in the response to drought stress, nutritional deficiencies, and copper homeostasis (94). Cao *et al.* (90) presumed that zma-miR397 can facilitate the oxidative stress response by upregulating the expression of laccases in rubredoxin. This mechanism enables it to counteract drought stress by regulating the dynamic balance of oxidation, thereby preventing damage to the cell membrane. Zm00001d012161 and Zm00001d042525 contribute to GO:0010038 (response to metal ion) while Zm00001d025784, Zm00001d037489 (rpl23a) and Zm00001d003127 are associated with GO:0006979 (response to oxidative stress), which indicates these genes may be regulated by zma-miR397, play roles in oxidation reaction and could improve the drought resistance of maize. These findings indicate these new regulations between genes and miRNAs offer new perspectives on the molecular mechanisms underlying drought tolerance and could potentially reveal novel targets for the breeding development of drought-tolerant maize varieties.
- *HetFCM can discover genes related to oil content of maize*.
  HetFCM can discover genes with single nucleotide polymorphisms (SNPs) related to oil content of maize. We download genetic loci information associated with oil content related traits from ZAEMAP (95) (http://www.zeamap.com/). As shown in Figure 8, we set the threshold of gene numbers to 10 and identify two co-modules, co-module 9 and co-module 26, which are significantly associated with oil content.
  In co-module 9, SNP chr9.s_108539819 (*p*-value=6.6711 × 10^−6^) is located at Zm00001d046865 (cer2), while Zm00001d046865 is annotated with GO:0006633 (fatty acid biosynthetic process) and GO:1901570 (fatty acid derivative biosynthetic process). Studies have also shown that Zm00001d046865 is involved in the synthesis of fatty acids (96). Fatty acid is the main component of maize oil, and the molecular mechanism of maize oil production can be analyzed by exploring the genes related to fatty acid synthesis, which can promote the breeding of maize varieties (97). According to the statistical results, co-module 26 contains the most statistically significant SNPs associated with oil content, and we conjecture that co-module 26 is the most relevant to oil content. In co-module 26, SNP chr6.s_108317526 (*p*-value=7.2199 × 10^−6^) is located at Zm00001d047186 (pco145462), which is annotated by *40S ribosomal protein S24, putative, expressed*. 40S ribosomal protein S24 is associated with oil content (98). Zm00001d002768 (ole1), in co-module 26, is a highly representative oleosin gene, which represents 75% of all the oleosin transcripts in the transcriptome at 21 days after pollination (99). Zm00001d036982 (ln1) encodes diacylglycerol acyltransferase (DGAT1-2), catalyzes the final step of oil synthesis (98) and is associated with linoleic acid. Zm00001d036985 is in co-module 26, and it is located on chromosome 6 with Zm00001d036982. The physical distance between the two genes is 98 173 base sites, which indicates that Zm00001d036985 and Zm00001d036982 are physically close to each other, thus Zm00001d036985 and Zm00001d036982 may have similar functions and Zm00001d036985 may also be related to oil content. These results prove that HetFCM can detect functional molecular modules related to oil content and provide new insights for crop breeding.

## Online system plantform

**Figure 9.**
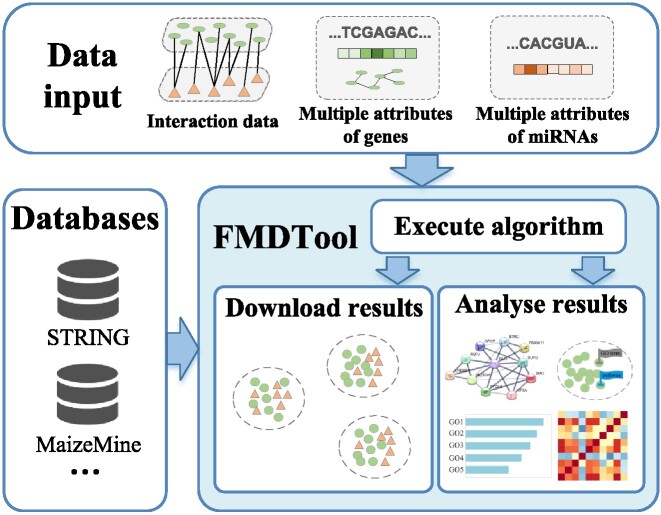
The overall architecture of FMDTool (Functional-Module Detection Tool) mainly consists of three parts: algorithm execution, result download and analysis.

Several co-module detection methods provide accessible code for offline usages, which faciliate the methodology comprehension and improvement, but impose a formidable challenge for biologists to conveniently configure and use them. Therefore, we designed a user-friendly functional module detection and analysis online tool (**FMDTool**) and shared it at http://www.sdu-idea.cn/FMDTool. As illustrated in Figure 9, given the corresponding data, FMDTool can identify functional modules (and co-modules) of any species. Meanwhile, it provides analysis services on Human and Maize datasets, such as annotating molecules in modules, visualizing gene interaction networks and enrichment analysis. HetFCM integrated into FMDTool can effectively fuse interplays and multi-type attributes of different molecules to detect co-modules with two types of molecules, which distinguishes it from the majority of existing methods. In addition, the uploaded data and returned results will be automatically deleted within three days after the completion of the algorithm, so the user has enough time to download the results and data security can be ensured.

## Discussion and conclusion

Functional module detection in heterogeneous molecular networks is a fundamental and important problem in system biology, which reveals complex regulatory relations between heterogeneous molecules and organizes the network into several small parts with specific functions. Many researchers have shown that the detection of co-modules of heterogeneous molecules can systematically reveal the genetic mechanism of phenotypes, which holds paramount implications for cancer diagnosis and treatment, drug development, and crop breeding (2,12,15,23,100).

In this study, we present HetFCM, a powerful multi-omics data integration tool, to detect molecules associated with phenotypes and mine reliable functional co-modules. We take the gene–miRNA co-module detection as an example for analysis. HetFCM achieves the best performance at both molecule and module levels compared to existing methods. We apply HetFCM to two important tasks: human cancer and plant phenotype. In these applications, HetFCM finds out modules with strong molecular relationships, remarkable expression patterns, and significant function enrichment. In the human breast cancer study, we discover gene–miRNA co-modules related to pancarcinoma and breast cancer and significantly enriched pathways, such as P53 signaling pathway and cell cycle. The detected co-modules also contain some potential gene–miRNA interactions, which offer novel standpoints on regulatory mechanisms. The co-modules detected by HetFCM may contain the cooperative driver pathways, which interact and synergistically trigger cancer. We detect the co-modules potentially associated with breast cancer, even though they have not been enriched into breast cancer based on the existing GO annotations. HetFCM can also find out co-modules associated with cancer subtypes. More importantly, the effectiveness of co-modules has been validated in clinical data from breast cancer. As for the results of maize breeding, we identify several co-modules related to lipid storage, drought tolerance and oil content, thereby offering new perspectives to breeding experts. HetFCM not only can effectively integrate new omics data with good expandability but also can be easily extended to identify multi-layer functional modules, such as gene–miRNA–lncRNA triple-layer modules, revealing more complex genetic mechanisms of molecules. In conclusion, these experimental results prove that HetFCM has a powerful multi-omics data integration capability to mine meaningful co-modules, it provides new insights for molecular mechanisms of pathogenic and phenotypic generation.

Although HetFCM achieves good performance, it still has several limitations. First, HetFCM needs users to collect and organize multi-source omics data. However, HetFCM can work with only molecular expression and interaction data, for its good expandability. Second, the time complexity of HetFCM is marginally higher than single-omics data-based methods, but it can effectively model interplays and multi-type attributes of different molecules. In our future work, we will discover dynamic co-modules based on spatiotemporal multi-omics data to reveal the dynamic regulatory process of living systems, and explain the pathogenesis and phenotypic mechanism.

## Supplementary Material

gkad1174_supplemental_files

## Data Availability

The code for HetFCM is available at http://www.sdu-idea.cn/FMDTool.
