# Supplementary material for: HetFCM: functional co-module discovery by heterogeneous network co-clustering
Source: Nucleic Acids Res. 2023 Dec 13;52(3):e16. doi: 10.1093/nar/gkad1174 (PMC10853805; doi:10.1093/nar/gkad1174)
Supplement: gkad1174_supplemental_files [file gkad1174_supplemental_files.zip › HetFCM_supl.pdf]

Published online DD MM YYYY *Nucleic Acids Research, YYYY, Vol. xx, No. xx* **1–13**
doi:10.1093/nar/gkn000

Supplementary materials of 'HetFCM: Functional co-module discovery by heterogeneous network co-clustering'

Haojiang Tan [1,2], Maozu Guo [3], Jian Chen [4], Jun Wang [2,*], and Guoxian Yu [1,2,*]

[1]School of Software, Shandong University, Jinan, 250101, Shandong, China
[2]Joint SDU-NTU Centre for Artificial Intelligence Research, Shandong University, Jinan, 250101, China
[3]College of Electrical and Information Engineering, Beijing Uni. of Civil Eng. and Arch., Beijing, 100044, China
[4]College of Agronomy & Biotechnology, China Agricultural University, Beijing, 100193, China

1 DATASET

Table S1 lists the collected molecular interaction data and multi-omics data to realize co-module detection. The details of these datasets are as follows.

For Human dataset, we first download the expression profiles of genes and miRNAs from TCGA database, obtain 11,621 genes and 1,602 miRNAs across 1,089 samples and denote them by $\mathbf{X}_{11}$ and $\mathbf{X}_{21}$, respectively. Then, we download the 60,900 known interactions between 11,621 genes and 1,602 miRNAs from miRTarBase database (1) and denote them by $\mathbf{W}_{12}$. In addition, we download protein-protein interaction data from STRING database (2), map proteins to genes, reserve the maximum protein-protein interaction value as the gene-gene interaction value, and obtain gene-gene interaction data represented by $\mathbf{X}_{12}$. Finally, we download gene sequence data and miRNA sequence data and denote them by $\mathbf{X}_{13}$ and $\mathbf{X}_{22}$, respectively.

For Maize dataset, we first download sequence data containing 35,748 genes and 344 miRNAs from MaizeGDB (https://www.maizegdb.org/) and denote them by $\mathbf{X}_{13}$ and $\mathbf{X}_{22}$, respectively. Then, we download the 4,291 known interactions between the above 35,748 genes and 344 miRNAs from PmiREN database (3) and denote them by $\mathbf{W}_{12}$. In addition, we download protein-protein interaction data from STRING database (2), convert them to the gene-gene interaction as the same approach on Human dataset, and obtain gene-gene interaction data represented by $\mathbf{X}_{12}$. Finally, we download the expression profiles of genes and miRNAs from NCBI (GSE136087) (4) and denote them by $\mathbf{X}_{11}$ and $\mathbf{X}_{21}$, respectively.

2 DETAILS OF VGAE

Variational Graph Auto-Encoder (VGAE) (5) is an unsupervised learning framework for network-structured data based on the variational auto-encoder (6). It can incorporate molecular attribute data and interaction data by graph convolutional network (GCN) (5) to learn the molecular embedding from the perspective of data distribution, reconstruct and complement the sparse interaction data. The VGAE mainly includes two parts, the inference part and the generative part. In the inference part, it takes adjacency matrix $\mathbf{A}$ and molecular attribute $\mathbf{H}_i$ as input and obtains a latent variable $\mathbf{Z}_i$ (also called the molecular embedding) as output, while in the generative part, the reconstructed adjacency matrix $\mathbf{A}_i^{'}$ is obtained according to the molecular embedding $\mathbf{Z}_i$.

Inference part: VGAE uses an inference model parameterized by a two-layer GCN to obtain the molecular embedding $\mathbf{Z}_i$:

$$\begin{aligned} q(\mathbf{Z}_i|\mathbf{H}_i,\mathbf{A}) = \prod\nolimits_{j=1}^{g+m} q(\mathbf{z}_j|\mathbf{H}_i,\mathbf{A}) \\ \text{with } q(\mathbf{z}_j|\mathbf{H}_i,\mathbf{A}) = \mathcal{N}(\mathbf{z}_j|\boldsymbol{\mu}_j, diag(\boldsymbol{\sigma}_j^2)) \end{aligned} \tag{S1}$$

where $\boldsymbol{\mu} = \mathrm{GCN}_{\boldsymbol{\mu}}(\mathbf{H}_i,\mathbf{A})$ is the matrix of mean vectors $\boldsymbol{\mu}_j$ and $\log\boldsymbol{\sigma} = \mathrm{GCN}_{\boldsymbol{\sigma}}(\mathbf{H}_i,\mathbf{A})$ is the matrix of the logarithm standard deviation vectors $\log\boldsymbol{\sigma}_j$. The logarithmic standard deviation can capture the data distribution uncertainty in the underlying space. $\mathcal{N}(\mathbf{z}|\boldsymbol{\mu},\boldsymbol{\sigma}^2)$ means that $\mathbf{z}$ is normally distributed with mean $\boldsymbol{\mu}$ and standard deviation $\boldsymbol{\sigma}$. g and m are the numbers of genes and miRNAs, respectively. The two-layer GCN is defined as follows:

$$\begin{aligned} \mathrm{GCN}(\mathbf{H}_i,\mathbf{A}) = \tilde{\mathbf{A}}\mathrm{ReLU}(\tilde{\mathbf{A}}\mathbf{H}_i\mathbf{W}_0)\mathbf{W}_1 \\ \tilde{\mathbf{A}} = \mathbf{D}^{-1/2}\mathbf{A}\mathbf{D}^{-1/2} \end{aligned} \tag{S2}$$

where $\{\mathbf{W}_i\}_{i=0}^{1}$ is the weight matrix, $\tilde{\mathbf{A}}$ is the symmetrically normalized adjacency matrix, $\mathbf{D}$ is the degree matrix of $\mathbf{A}$ and $\mathrm{ReLU}(\cdot) = max(0,\cdot)$.

Generative part: The generative part defines inner product between molecular embedding $\mathbf{Z}_i$ as a reconstructed adjacency matrix as follows:

$$\mathbf{A}_i^{'} = \sigma(\mathbf{Z}_i,\mathbf{Z}_i^T) \tag{S3}$$

where $\sigma(\cdot)$ is the logistic Sigmoid function.

*To whom correspondence should be addressed. Tel: +86 531 88391516; Fax: +86 531 88391686; Email: {gxyu, kingjun}@sdu.edu.cn.

© YYYY The Author(s)
This is an Open Access article distributed under the terms of the Creative Commons Attribution Non-Commercial License (http://creativecommons.org/licenses/by-nc/2.0/uk/) which permits unrestricted non-commercial use, distribution, and reproduction in any medium, provided the original work is properly cited.

Table S1. Details of collected data sources.

Species	Datasets	Size	Notation	Source
Human	gene-miRNA interaction	11,621×1,602	$\mathbf{W}_{12}$	miRTarBase database (1)
	gene expression profile	11,621×1,089	$\mathbf{X}_{11}$	https://www.cancer.gov/about-nci/organization/ccg/research/structural-genomics/tcga
	gene-gene interaction	11,621×11,621	$\mathbf{X}_{12}$	STRING database (2)
	gene sequence	11,621×256	$\mathbf{X}_{13}$	https://uswest.ensembl.org/info/data/index.html
	miRNA expression profile	1,602×1,089	$\mathbf{X}_{21}$	https://www.cancer.gov/about-nci/organization/ccg/research/structural-genomics/tcga
	miRNA sequence	1,602×256	$\mathbf{X}_{22}$	https://uswest.ensembl.org/info/data/index.html
Maize	gene-miRNA interaction	35,748×344	$\mathbf{W}_{12}$	PmiREN database (3)
	gene expression profile	35,748×36	$\mathbf{X}_{11}$	NCBI (GSE136087) (4)
	gene-gene interaction	35,748×35,748	$\mathbf{X}_{12}$	STRING database (2)
	gene sequence	35,748×256	$\mathbf{X}_{13}$	http://www.maizegdb.org/
	miRNA expression profile	344×36	$\mathbf{X}_{21}$	NCBI (GSE136087) (4)
	miRNA sequence	344×256	$\mathbf{X}_{22}$	http://www.maizegdb.org/

The generative part can be represented as follows:

$$p(\mathbf{A}|\mathbf{Z}_i)=\prod_{i=1}^{g+m}\prod_{j=1}^{g+m}p(\mathbf{A}_{ij}|\mathbf{z}_i,\mathbf{z}_j)$$
$$\text{with } p(\mathbf{A}_{ij}=1|\mathbf{z}_i,\mathbf{z}_j)=\sigma(\mathbf{z}_i^T,\mathbf{z}_j) \quad \text{(S4)}$$

Loss function: The loss function of the VGAE includes two parts, the first part is the binary cross-entropy loss between the input adjacency matrix $\mathbf{A}$ and the reconstructed adjacency matrix $\mathbf{A}_i^{'}$, which can be represented by Eq. (S4), while the second part is the Kullback-Leibler divergence between $q(\mathbf{Z}_i|\mathbf{H}_i,\mathbf{A})$ and the Gaussian prior $p(\mathbf{Z}_i)$:

$$p(\mathbf{Z}_i)=\prod_{j=1}^{g+m}\mathcal{N}(\mathbf{z}_j|0,\mathbf{I}) \quad \text{(S5)}$$

where $\mathbf{I}$ is a diagonal matrix with value 1. As a result, we obtain the loss of VGAE:

$$\mathcal{L}=E_{q(\mathbf{Z}_i|\mathbf{H}_i,\mathbf{A})}[\log p(\mathbf{A}|\mathbf{Z}_i)]-\mathrm{KL}(q(\mathbf{Z}_i|\mathbf{H}_i,\mathbf{A})||p(\mathbf{Z}_i)) \quad \text{(S6)}$$

By optimizing Eq. (S6), we can fuse the attribute data $\mathbf{H}_i$ as well as association data $\mathbf{A}$ of molecules to obtain the molecular embedding $\mathbf{Z}_i$ and reconstruct the adjacency matrix $\mathbf{A}_i^{'}$ by Eq. (S3).

3 OPTIMIZATION OF THE OBJECTIVE FUNCTION

In this section, we iteratively optimize the objective function Eq. (S7) of HetFCM with respect to one variable while fixing the other variables until convergence:

$$J=\sum_{i=1}^{v}\mathbf{w}_i\left\|\mathbf{W}_i-\mathbf{G}_1\mathbf{S}_i\mathbf{G}_2^T\right\|_F^2+\alpha\sum_{i=1,2}\|\mathbf{X}_{i1}-\mathbf{G}_i\mathbf{B}\|_F^2$$
$$+\beta\sum_{i=1,2}\left\|\mathbf{G}_i^T\mathbf{G}_i-\mathbf{I}\right\|_F^2+\gamma\|\mathbf{w}\|_F^2$$
$$\text{s.t. } \mathbf{G}_i\geqslant 0,\mathbf{S}_i\geqslant 0,\mathbf{w}^T\mathbf{1}=1,\mathbf{w}_i\geqslant 0 \quad \text{(S7)}$$

Updating $\mathbf{S}_i$

Optimizing Eq. (S7) with respect to $\mathbf{S}_i$ is equivalent to optimizing

$$J(\mathbf{S}_i)=\sum_{i=1}^{v}\mathbf{w}_i\left\|\mathbf{W}_i-\mathbf{G}_1\mathbf{S}_i\mathbf{G}_2^T\right\|_F^2 \quad \text{(S8)}$$

Let $\frac{\partial J(\mathbf{S}_i)}{\partial \mathbf{S}_i}=0$ and we can obtain the following update formula

$$\mathbf{S}_i=(\mathbf{G}_1^T\mathbf{G}_1)^{-1}\mathbf{G}_1^T\mathbf{W}_i\mathbf{G}_2(\mathbf{G}_2^T\mathbf{G}_2)^{-1} \quad \text{(S9)}$$

Updating $\mathbf{G}_1$

Optimizing Eq. (S7) with respect to $\mathbf{G}_1$ is equivalent to optimizing

$$J(\mathbf{G}_1)=\sum_{i=1}^{v}\mathbf{w}_i\left\|\mathbf{W}_i-\mathbf{G}_1\mathbf{S}_i\mathbf{G}_2^T\right\|_F^2+\alpha\|\mathbf{X}_{11}-\mathbf{G}_1\mathbf{B}\|_F^2$$
$$+\beta\left\|\mathbf{G}_1^T\mathbf{G}_1-\mathbf{I}\right\|_F^2$$
$$\text{s.t. } \mathbf{G}_1\geqslant 0 \quad \text{(S10)}$$

For the constraint $\mathbf{G}_1\geq 0$, we adopt an iterative multiplicative updating solution. The corresponding Lagrange function is

$$\mathcal{L}(\mathbf{G}_1)=\sum_{i=1}^{v}\mathbf{w}_i\left\|\mathbf{W}_i-\mathbf{G}_1\mathbf{S}_i\mathbf{G}_2^T\right\|_F^2+\alpha\|\mathbf{X}_{11}-\mathbf{G}_1\mathbf{B}\|_F^2$$
$$+\beta\left\|\mathbf{G}_1^T\mathbf{G}_1-\mathbf{I}\right\|_F^2-tr(\mathbf{A}\mathbf{G}_1^T)$$
$$\text{s.t. } \mathbf{G}_1\geqslant 0 \quad \text{(S11)}$$

where $\mathbf{A}\in\mathbb{R}^{m\times k}$ and $\mathbf{A}\geq 0$ is the Lagrange multiplier for $\mathbf{G}_1\geq 0$. Let $\frac{\partial \mathcal{L}(\mathbf{G}_1)}{\partial \mathbf{G}_1}=0$ and we can obtain

$$\mathbf{A}=-2\mathbf{P}+2\mathbf{G}_1\mathbf{Q}$$
$$\mathbf{P}=\alpha\mathbf{X}_{11}\mathbf{B}^T+\sum_{i=1}^{v}\mathbf{w}_i\mathbf{W}_i\mathbf{G}_2\mathbf{S}_i^T+2\beta\mathbf{G}_1 \quad \text{(S12)}$$
$$\mathbf{Q}=\alpha\mathbf{B}\mathbf{B}^T+\sum_{i=1}^{v}\mathbf{w}_i\mathbf{S}_i\mathbf{G}_2^T\mathbf{G}_2\mathbf{S}_i^T+2\beta\mathbf{G}_1^T\mathbf{G}_1$$

According to Karush-Kuhn-Tucker (KKT) condition $\mathbf{A}_{ij}[\mathbf{G}_1]_{ij}=0$, we get $[-2\mathbf{P}+2\mathbf{G}_1\mathbf{Q}]_{ij}[\mathbf{G}_1]_{ij}=0$. Introducing $\mathbf{P}=\mathbf{P}^+-\mathbf{P}^-$ and $\mathbf{Q}=\mathbf{Q}^+-\mathbf{Q}^-$, where $\mathbf{P}_{ij}^+=(|\mathbf{P}_{ij}|+\mathbf{P}_{ij})/2$ and $\mathbf{P}_{ij}^-=(|\mathbf{P}_{ij}|-\mathbf{P}_{ij})/2$, we get $[-\mathbf{P}^++\mathbf{P}^-+\mathbf{G}_1\mathbf{Q}^+-\mathbf{G}_1\mathbf{Q}^-]_{ij}[\mathbf{G}_1]_{ij}=0$. Finally, we obtain the update formula for $\mathbf{G}_1$ as follows:

$$[\mathbf{G}_1]_{ij}\leftarrow[\mathbf{G}_1]_{ij}\sqrt{\frac{[\mathbf{P}^++\mathbf{G}_1\mathbf{Q}^-]_{ij}}{[\mathbf{P}^-+\mathbf{G}_1\mathbf{Q}^+]_{ij}}} \tag{S13}$$

Updating $\mathbf{G}_2$

Similar to updating $\mathbf{G}_1$, the update formula for $\mathbf{G}_2$ is

$$[\mathbf{G}_2]_{ij}\leftarrow[\mathbf{G}_2]_{ij}\sqrt{\frac{[\mathbf{U}^++\mathbf{G}_2\mathbf{V}^-]_{ij}}{[\mathbf{U}^-+\mathbf{G}_2\mathbf{V}^+]_{ij}}} \tag{S14}$$

where $\mathbf{U}=\alpha\mathbf{X}_{21}\mathbf{B}^T+\sum_{i=1}^{v}\mathbf{w}_i\mathbf{B}_i^T\mathbf{G}_1\mathbf{S}_i+2\beta\mathbf{G}_2$ and $\mathbf{V}=\alpha\mathbf{B}\mathbf{B}^T+\sum_{i=1}^{v}\mathbf{w}_i\mathbf{S}_i^T\mathbf{G}_1^T\mathbf{G}_1\mathbf{S}_i+2\beta\mathbf{G}_2^T\mathbf{G}_2$.

Updating $\mathbf{B}$

Similar to updating $\mathbf{G}_1$, the update formula for $\mathbf{B}$ is

$$\mathbf{B}_{ij}\leftarrow\mathbf{B}_{ij}\sqrt{\frac{[\mathbf{C}^++\mathbf{D}^-\mathbf{B}]_{ij}}{[\mathbf{C}^-+\mathbf{D}^+\mathbf{B}]_{ij}}} \tag{S15}$$

where $\mathbf{C}=\sum_{i=1,2}\mathbf{G}_i^T\mathbf{X}_{i1}$ and $\mathbf{D}=\sum_{i=1,2}\mathbf{G}_i^T\mathbf{G}_i$.

Updating $\mathbf{w}$

Optimizing Eq. (S7) with respect to $\mathbf{w}$ is equivalent to optimizing

$$\begin{aligned}&J(\mathbf{w})=\sum_{i=1}^{v}\mathbf{w}_i\|\mathbf{W}_i-\mathbf{G}_1\mathbf{S}_i\mathbf{G}_2^T\|_F^2+\gamma\|\mathbf{w}\|_F^2\\&\text{s.t.}\mathbf{w}_i\geq0,\mathbf{w}^T\mathbf{1}=1\end{aligned} \tag{S16}$$

For simplicity, let $\mathbf{b}_i=\|\mathbf{W}_i-\mathbf{G}_1\mathbf{S}_i\mathbf{G}_2^T\|_F^2$, we need to solve the following problem

$$\begin{aligned}&J(\mathbf{w})=\mathbf{w}^T\mathbf{b}+\gamma\mathbf{w}^T\mathbf{w}\\&\text{s.t.}\mathbf{w}_i\geq0,\mathbf{w}^T\mathbf{1}=1\end{aligned} \tag{S17}$$

The corresponding Lagrange function is

$$\mathcal{L}(\mathbf{w},\eta,\lambda)=\mathbf{w}^T\mathbf{b}+\gamma\mathbf{w}^T\mathbf{w}-\eta(\mathbf{w}^T\mathbf{1}-1)-\lambda^T\mathbf{w} \tag{S18}$$

where η, $\lambda\in\mathbb{R}^{v\times1}$ and $\lambda_i\geq0$ are the Lagrange multipliers. Taking the derivative of Eq. (S18) with respect to $\mathbf{w}$ and setting it to zero, we have

$$\mathbf{b}+2\gamma\mathbf{w}-\eta\mathbf{1}-\lambda=\mathbf{0} \tag{S19}$$

Then for $\mathbf{w}_i$, we have

$$\mathbf{b}_i+2\gamma\mathbf{w}_i-\eta-\lambda_i=0 \tag{S20}$$

Note that $\mathbf{w}_i\lambda_i=0$ according to the KKT condition, then we have

$$\mathbf{w}_i=(\frac{1}{2\gamma}\eta-\frac{1}{2\gamma}\mathbf{b}_i)_+ \tag{S21}$$

where $(v)_+=max(0,v)$. We define the following function

$$g_i(\eta)=\sum_{i=1}^{v}(\frac{1}{2\gamma}\eta-\frac{1}{2\gamma}\mathbf{b}_i)_+-1 \tag{S22}$$

According to Eqs (S21)-(S22) and the constraint $\mathbf{w}^T\mathbf{1}=1$, we have the following equation

$$g_i(\eta)=0 \tag{S23}$$

Consequently, the value of η is the root of function $g_i(x)$. Since $g_i(x)$ is a piecewise linear and monotonically increasing function, thus the root can be easily obtained by Newton's method. After calculating η, the optimal solution to the problem Eq. (S16) can be obtained by Eq. (S21).

4 THE TIME COMPLEXITY ANALYSIS OF HETFCM

The time complexity of HetFCM comes from two sources, the complexity of gene-miRNA association matrices calculation and that of co-clustering. For the former, it involves multiple VGAE. VGAE includes the inference part and the generative part. A two-layer GCN parameterizes the inference part. The time complexity of GCN is $O(l\|\mathbf{A}\|_0f+lhf^2)$, where l is number of layers, h is number of nodes ($h=g+m$), $\|\mathbf{A}\|_0$ is number of nonzeros in the adjacency matrix, and f is number of features. The generative part defines the inner product with the time complexity $O(h^2)$. For the latter, it refers to the calculation of $\mathbf{G}_1$, $\mathbf{G}_2$, $\mathbf{S}_i$, $\mathbf{B}$ and $\mathbf{w}$. The time complexity of these terms is $O(gk^2)$, $O(mk^2)$, $O(vgmk)$, $O((g+m)nk)$ and $O(v)$ respectively. Although the efficiency of HetFCM is not the best compared to single omics data-based co-modules detection methods, it can effectively model interplays and multi-type attributes of different molecules. Compared with the competitive methods, the proposed HetFCM achieves the best performance under acceptable runtime. In addition, when the adjacency matrix $\mathbf{A}$ is sparser, its time complexity can be greatly reduced.

5 DEFINITION OF EVALUATION INDICATORS

Modularity reflects the closeness between genes and miRNAs in the co-module. The higher the modularity value of the detected gene-miRNA module, the stronger the correlation of genes and miRNAs in this module. We adopt the following modularity metric (7):

$$modularity(M)=\frac{\sum_{i\in I,j\in J}\mathbf{W}_{12}(i,j)}{|\mathcal{I}|\cdot|\mathcal{J}|} \tag{S24}$$

where $\mathbf{W}_{12}(i,j)$ is the element at row i and column j of $\mathbf{W}_{12}$, $\mathcal{I}$ and $\mathcal{J}$ are the gene set and miRNA set in the gene-miRNA

module M, respectively. We use modularity to evaluate the compactness of co-module network topology.

To test the statistical significance of gene-miRNA co-modules, we randomly select 1,000 co-modules and calculate the modularity by Eq. (S24), then use Wilcoxon rank sum test to calculate the p-value of co-modules detected by each method. We use p-value to quantify the statistical significance, and the p-value smaller than 0.01 indicates the detected gene-miRNA co-modules have significant topology (8, 9).

Enrichment rate (ER) is an important indicator to measure the degree of molecular enrichment in the module (M). Enrichment rate function is defineed as following:

$$ER(M)=\frac{N_{en}}{N} \tag{S25}$$

where N refers to the number of molecules in the module and N_{en} refers to the number of enriched molecules. We calculate the enrichment rate of gene modules as well as the randomly generated modules, and then use their ratio to select the optimal T_1 in Section 6.

Precision is defined to measure the degree of association between the module (M) and the phenotype as follows:

$$precision(M)=\frac{N_a}{N} \tag{S26}$$

where N refers to the number of molecules in the module and N_a refers to the number of molecules associated with the phenotype. We use *Precision* to evaluate the association degree between the modules (co-modules and triple-layer modules) and phenotype (i.e., breast cancer).

6 PARAMETER ANALYSIS

There are seven parameters (k, α, β, γ, t, T_1 and T_2) in HetFCM. k is the number of co-clusters. α controls the weight of molecular expression data. β is the hyperparameter that controls the orthogonality of $\mathbf{G}_1$ and $\mathbf{G}_2$. γ is the regularized term coefficient to control the weights of gene-miRNA association matrices. t is the number of retained top gene-miRNA association pairs among all unknown gene-miRNA associations. T_1 and T_2 are the thresholds for detecting gene and miRNA modules, respectively.

It is difficult to automatically determine the number of co-clusters k. To select the optimal parameter k, we utilize the K-means algorithm (10) for cluster analysis based on gene expression data. We vary $k\in\{20,25\cdots,50\}$ and report the sum of the squared distances from centroids (SSq distances from centroids) under different k. For Human dataset, as shown in Figure S1**A**, the line makes the sharpest bend (the elbow) when $k=30$, indicating that $k=30$ achieves optimal performance according to Elbow method (11). Therefore, we set $k=30$ for Human dataset. As for Maize dataset, we also select $k=30$ based on the Elbow method as shown in Figure S1**B**.

As to the parameters α and β, they were empirically determined by the values of each corresponding term of the objective function as shown in Eq. (S27) (12). Their values are shown in Figure S2**A-1** and S2**B-1**.

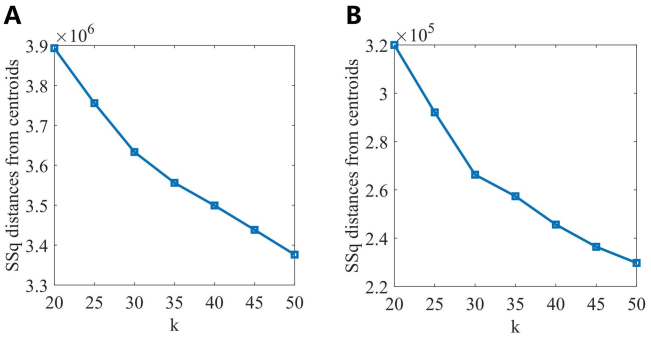

Figure S1. Results of cluster analysis under different values of k. **A** shows the sum of the squared distances (SSq distances) from centroids on Human dataset, while **B** shows SSq distances from centroids on Maize dataset.

$$\begin{aligned}L_1&=\sum_{i=1}^{v}\mathbf{w}_i\left\|\mathbf{W}_i-\mathbf{G}_1\mathbf{S}_i\mathbf{G}_2^T\right\|_F^2+\gamma\|\mathbf{w}\|_F^2\\ L_2&=\sum_{i=1,2}\|\mathbf{X}_{i1}-\mathbf{G}_i\mathbf{W}\|_F^2\\ L_3&=\sum_{i=1,2}\left\|\mathbf{G}_i^T\mathbf{G}_i-\mathbf{I}\right\|_F^2\\ L&=L_1+L_2+L_3\end{aligned} \tag{S27}$$

We balance the values of L_1, L_2 and L_3 using parameters α and β to determine the values of them. Finally, we set $\alpha=0.1$ and $\beta=450$ for Human dataset, $\alpha=0.2$ and $\beta=0.001$ for Maize dataset. We also run HetFCM with four different parameter combinations to test the robustness of the results under the current parameter setting. As shown in Figure S2**A-2** and S2**B-2**, we found that the modules detected by HetFCM under different parameter combination settings enrich numerous same GO terms, whose numbers are roughly same and the enrichment degree distributions are nearly uniform. These results indicate HetFCM is robust to α and β and the current setting of them is reasonable for module detection.

We analyze the impacts of different γ on model performance for Human dataset. Figure S3**A** reports the weights($\mathbf{w}$) assigned to v ($v=5$) gene-miRNA association matrices $\{\mathbf{W}_i\}_{i=1}^5$ under different γ, while Figure S3**B-C** gives the performance of HetFCM under different γ. When $\gamma\leq 5*10^6$, with the increase of γ, the model performance improves. This is because more gene-miRNA association matrices are selected and assigned with different weights, and the genetic information are adaptively fused. When γ further increases, all the gene-miRNA association matrices are assigned with nearly equal weights, which can not effectively differentiate diverse genetic information contained in gene-miRNA association matrices, leading to descending performance. The analysis results of γ further indicate that differentially fusing gene-miRNA association matrices can improve the model performance. Hence, we set $\gamma=5*10^6$ for Human dataset. The adaptively assigned weight w for gene-miRNA association matrices is influenced by the parameter γ. In this setting ($\gamma=5*10^6$), miRNA expression data is assigned with the highest weight, indicating it is

the most important for co-module detection. This may be because miRNA expression data include important regulatory information, which helps to enrich gene-miRNA associations and identify gene-miRNA co-modules. The AUROC, AUPRC and F1-score on Maize dataset cannot be obtained, due to the unavailability of specific maize phenotype information. Therefore, we also set $\gamma = 5*10^6$ for Maize dataset. To study the rationality for this setting, we conducted a comparative analysis with other four as shown in Figure S5**A** based on enrichment analysis. The results indicate that when $\gamma = 5*10^6$, HetFCM outperforms the other settings. Therefore, we set $\gamma = 5*10^6$ on Maize dataset.

We also analyze the parameter t, which is the number of retained top gene-miRNA association pairs among all unknown gene-miRNA associations. As shown in Figure S4, the performance of HetFCM is robust under different t on Human dataset. Without loss of generality, we set $t = 60{,}000$ for Human dataset. As for Maize dataset, we also set $t = 60{,}000$. To test the validity for this setting, we compared the enrichment analysis results on this setting with other four as shown in Figure S5**B**. The results show that when $t = 60{,}000$, HetFCM achieves better performance than other settings. Therefore, we set $t = 60{,}000$ on Maize dataset.

T_1 and T_2 are parameters directly related to clustering. We analyze their influence on clustering through the enrichment rate and module modularity. Their definitions were given in Section 5. To determine the appropriate T_1, the enrichment rate of gene modules with respect to GO biological process is calculated. For comparision, we also calculate the mean enrichment rate for 100 corresponding random modules. As shown in Figure S6**A-1** and **A-2**, HetFCM reaches to the optimal performance when $T_1 = 6$ and $T_2 = 8$ on Human dataset. Therefore, we set $T_1 = 6$ and $T_2 = 8$ for Human dataset. As for Maize dataset, we set $T_1 = 6$ and $T_2 = 4$ according to Figure S6**B-1** and **B-2**.

7 ANALYSIS ON MODULE LEVEL EVALUATION

As shown in Figure 2 of the main text, among the compared methods, TsRFR has the worst performance. That is because it obtains the regulatory network between miRNAs and genes using only expression data of gene and miRNA, which may lead to information loss due to the conversion from attribute data (expression data) to association data. In spite of the same data sources and the conversion from attribute data to network data, NetNMF performs better than TsRFR. This is because NetNMF constructs gene co-expression network, miRNA co-expression network and gene-miRNA co-expression network based on expression data. Such multiple uses of attribute data to construct different networks is conducive to alleviating information loss and achieving better performance. TsRFR only uses expression data once and cannot effectively deal with noise. For Pseudo_3d, we utilize the same method as NetNMF to construct three co-expression networks, and then obtain gene-miRNA co-modules by a bottom-up clustering strategy based on network. Because Pseudo_3d relies on the network topology and need sparse co-expression network, which leads to information loss, and thus its performance is worse than NetNMF. Among the remaining three methods, SNPLS, HOGMHC and JONMF incorporate molecular expression and network data by imposing network constraints. The modularity of SNPLS is lower than that of HOGMHC and JONMF, because SNPLS only uses the gene-gene interaction network, while HOGMHC and JONMF fuse the gene-miRNA association network, which provides more genetic information for module detection. HetFCM has the best performance, this is because it can effectively utilize interplays and multi-type attributes of different molecules to complete the cross-layer association matrices, which contain critical genetic information and can guide the detection of co-modules. The adaptive weighted co-clustering on association matrices and attribute data enables the HetFCM to differently integrate the association matrices and to accurately identify the gene-miRNA co-modules.

8 RESULTS ON MAIZE DATASET

Figure S7 reports the modularity and the GO terms enrichment degree of HetFCM and compared methods on Maize dataset. Compared to the results on Human dataset in Figure 2 of the main text, we can observe that the modularity of co-modules detected on Maize dataset is distinctly lower than that on Human dataset, due to the fact that the gene-miRNA interaction network of Maize dataset is significantly sparser than that of Human dataset. Other results yield similar conclusions to those obtained from Human dataset.

9 RESULTS OF ABLATION STUDY

To further explore the impact of each component in HetFCM, we set up four variants of HetFCM. HetFCM-w/oSeq, HetFCM-w/oGGI and HetFCM-w/oExp separately disregard molecular sequence data, gene-gene interaction and molecular expression data. These data are seperately excluded in the multi-omics data fusion section and not used by VGAE model. We also setup HetFCM-w/oHet, which directly excludes the objective function $\mathcal{O}_1$, namely diregard the important gene-miRNA interactions.

As shown in Figure S8**A** and **B**, HetFCM achieve the best modularity than its variants on Human dataset. These results indicate that these multi-omics attribute data of genes and miRNAs can complete the gene-miRNA associations, improve the modularity of the co-modules, and make the modules more dense in topology. When multiple gene-miRNA association matrices are ignored, the modularity of HetFCM-w/oHet decreases more seriously than that of the other three variants, which proves the adaptive weighted co-clustering can effectively capture the structural information of heterogeneous molecules and improve the modularity of modules.

According to Figure S8**C**, after removing the molecular sequence, the gene-gene interaction network, the molecular expression data and multiple gene-miRNA association matrices, the number of siginificant gene-miRNA bilayer modules detected by different variants on Human dataset decrease by 4, 7, 6 and 8, respectively. These results prove that multi-omics attribute data and heterogeneous network are beneficial to module detection and can make the function of the detected modules more significant. The results on Maize dataset give similar conclusions as that on Human dataset as shown in Figure S9.

10 WIKIPATHWAYS ENRICHED BY THE GENE-MIRNA CO-MODULES

The gene-miRNA co-modules detected by HetFCM can be enriched into various WikiPathways. Figure S10 represents WikiPathway WP2446 (retinoblastoma gene in cancer) enriched by the gene layer of co-module 2. Figure S11 represents WikiPathway WP2261 (glioblastoma signaling pathways) enriched by the miRNA layer of co-module 14. Figure S12 represents WikiPathway WP3929 (chemokine signaling pathway) enriched by co-module 20. These results indicate that the gene-miRNA co-modules detected by HetFCM can capture the key driver genes in the pathways, which further demonstrates the effectiveness of HetFCM.

11 LITERATURE VERIFICATION

To prove that the co-modules detected by HetFCM are accurate, we perform biomedical literature search on PubMed (13). For example, in gene-miRNA co-module 5, KIF23 is a member of kinesin family, which includes microtubule-dependent molecular motors that transport organelles within cells and move chromosomes during cell division. Alternative splicing of this gene results in multiple transcript variants. Recent literature indicates KIF23 plays an important role in the proliferation and migration of malignant cancer cells (14), and promotes triple negative breast cancer through activating epithelial-mesenchymal transition (15). Hsa-mir-106b is an miRNA that regulates the expression of KIF23 cell cycle progression (16). Yi *et al.* (17) used RNAhybrid, Venn and UpSetR plot and network analysis to construct a circRNA-miRNA-hubgenes network consisting of 5 circRNAs, 2 miRNAs (**hsa-miR-106b** and hsa-miR-5b) and 7 mRNAs (**KIF23**, **RRM2**, **CEP55**, CHEK1, **RACGAP1**, **ATAD2** and **KIF11**), where the molecules with bold font are also present in gene-miRNA co-module 5.

Although co-module 17 is not enriched in breast cancer directly, it is enriched in GO:0033598 (mammary gland epithelial cell proliferation), which is closely associated with breast cancer (18). As shown in Figure S13**A**, ESR1 and CCND1 are hub genes in co-module 17, and they interact with ZNF703. ESR1 (Estrogen receptor alpha) gene amplification is frequent in breast cancer (19) while CCND1 amplification is a prognostic factor for patients with estrogen receptor positive breast cancer (20). ZNF703 is a common Luminal B breast cancer oncogene that differentially regulates luminal and basal progenitors in human mammary epithelium (21). These three genes are associated with breast cancer. According to network propagation and random walk theory (22), hsa-let-7b may interact with ESR1, KRT19 and XBP1. Hsa-let-7b is associated with breast cancer (23), while ESR1, KRT19 and XBP1 are also related to breast cancer (19, 24, 25). Therefore, we infer that hsa-let-7b may have interactions with these three genes. These examples confirm that the cooperative interplays between genes and miRNAs in the co-modules detected by HetFCM not only have an important influence on the trigger and development of cancer, but also provide new insights for molecular mechanism analysis.

12 MORE DETAILS ON GENE-MIRNA-LNCRNA TRIPLE-LAYER MODULES

We utilize the same gene-related data as the gene-miRNA co-module detection to identify the gene-lncRNA co-modules. For this purpose, we download lncRNA expression data and lncRNA sequence data from the same sources as the gene-related data. Additionally, we download gene-lncRNA interaction data between 11,621 genes and 1,139 lncRNAs from RNAInter database (26), containing 16,198 interactions.

To show the intro/inter associations between molecules in the gene-miRNA-lncRNA triple-layer modules, we performed a visual association analysis based on the data sources listed in Table S2. As shown in Figure 6 of the main text, the two gene-miRNA-lncRNA triple-layer modules have dense topology and the key molecules in modules are significantly associated with breast cancer. These results suggest that HetFCM can be easily extended to detect multi-layer functional modules.

Table S2. The data sources for detecting gene-miRNA-lncRNA triple-layer modules.

Type	Source
gene-gene association	STRING database (2)
miRNA-miRNA association	co-expression networks based on PCC with value > 0.5
lncRNA-lncRNA association	co-expression networks based on PCC with value > 0.5
gene-miRNA association	miRTarBase database (1)
gene-lncRNA association	RNAInter database (26)
miRNA-lncRNA association	RNAInter database (26)

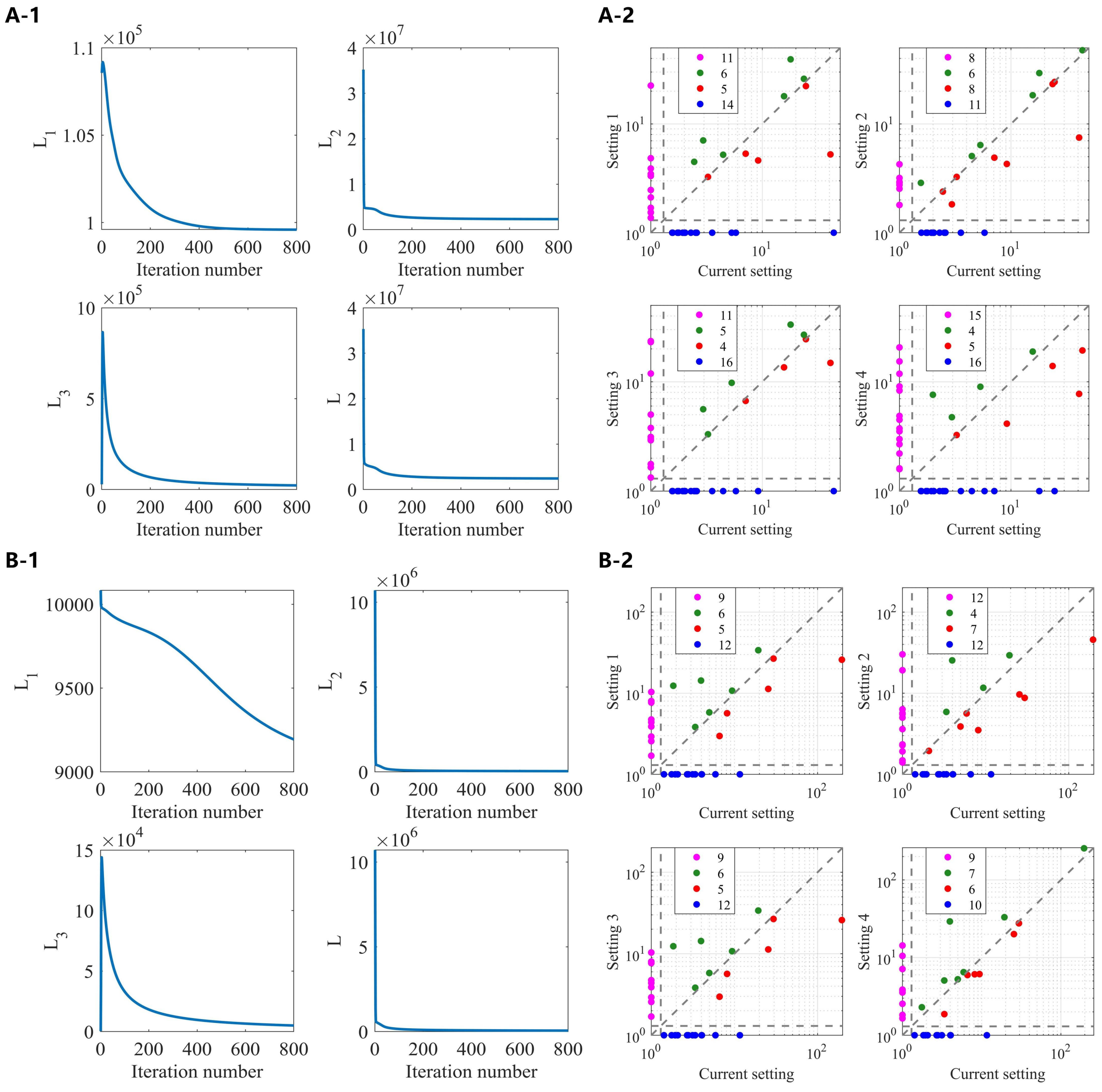

Figure S2. Parameter sensitivity analysis of α and β. **A-1** reports the objective values for L_1, L_2, L_3 and L on Human dataset. **A-2** compares current parameter setting ($k=30$, $\alpha=0.1$ and $\beta=450$) with other four based on enrichment analysis on Human dataset. The four settings are ($k=30$, $\alpha=1$ and $\beta=450$), ($k=30$, $\alpha=0.1$ and $\beta=900$), ($k=30$, $\alpha=1$ and $\beta=900$) and ($k=40$, $\alpha=0.1$ and $\beta=450$), respectively. **B-1** reports objective values for L_1, L_2, L_3 and L on Maize dataset. **B-2** compare the current setting ($k=30$, $\alpha=0.2$ and $\beta=0.001$) with other four based on enrichment analysis on Maize dataset. The four settings are ($k=30$, $\alpha=2$ and $\beta=0.001$), ($k=30$, $\alpha=0.2$ and $\beta=0.01$), ($k=30$, $\alpha=2$ and $\beta=0.01$) and ($k=40$, $\alpha=0.2$ and $\beta=0.001$), respectively.

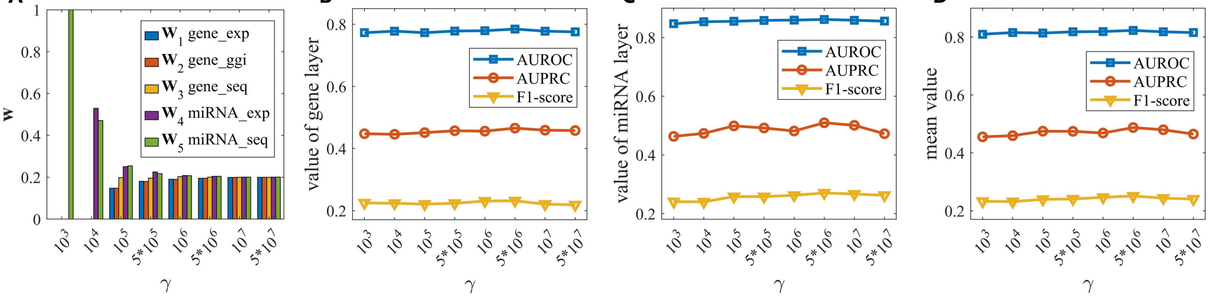

Figure S3. Results of HetFCM under different input values of γ on Human dataset. **A** reports weights ($\mathbf{w}$) assigned to 5 association matrices $\{\mathbf{W}_i\}_{i=1}^5$ under different γ. **B** gives evaluation metric values under different γ at gene level, while **C** gives the values at miRNA level. **D** reveals the mean values of evaluation metric under different γ at gene level and miRNA level.

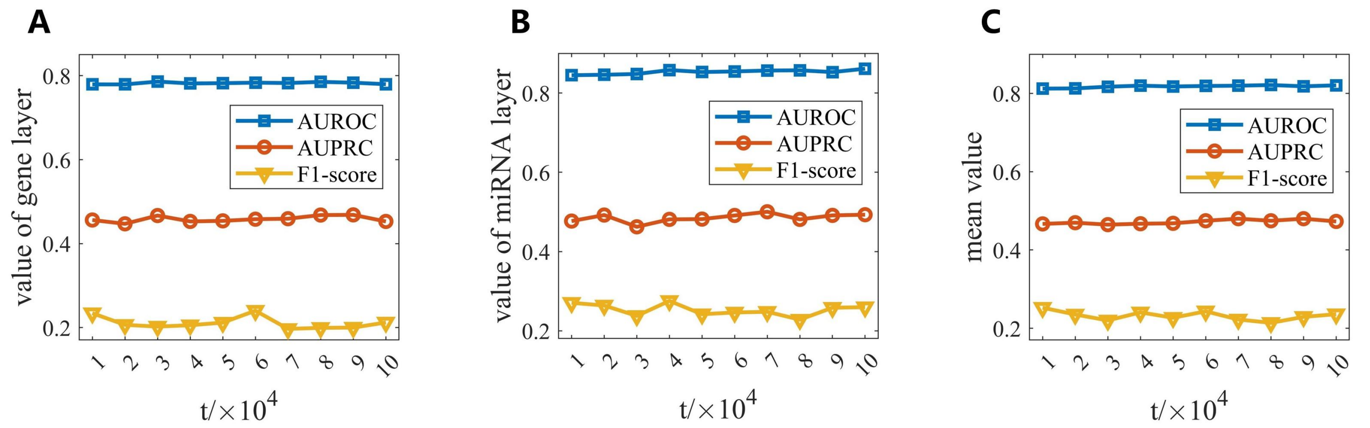

Figure S4. Results of HetFCM under different input values of t on Human dataset. **A** reports evaluation metric values under different t at gene level, while **B** reports the values at miRNA level. **C** gives the mean values of evaluation metric under different t at gene level and miRNA level.

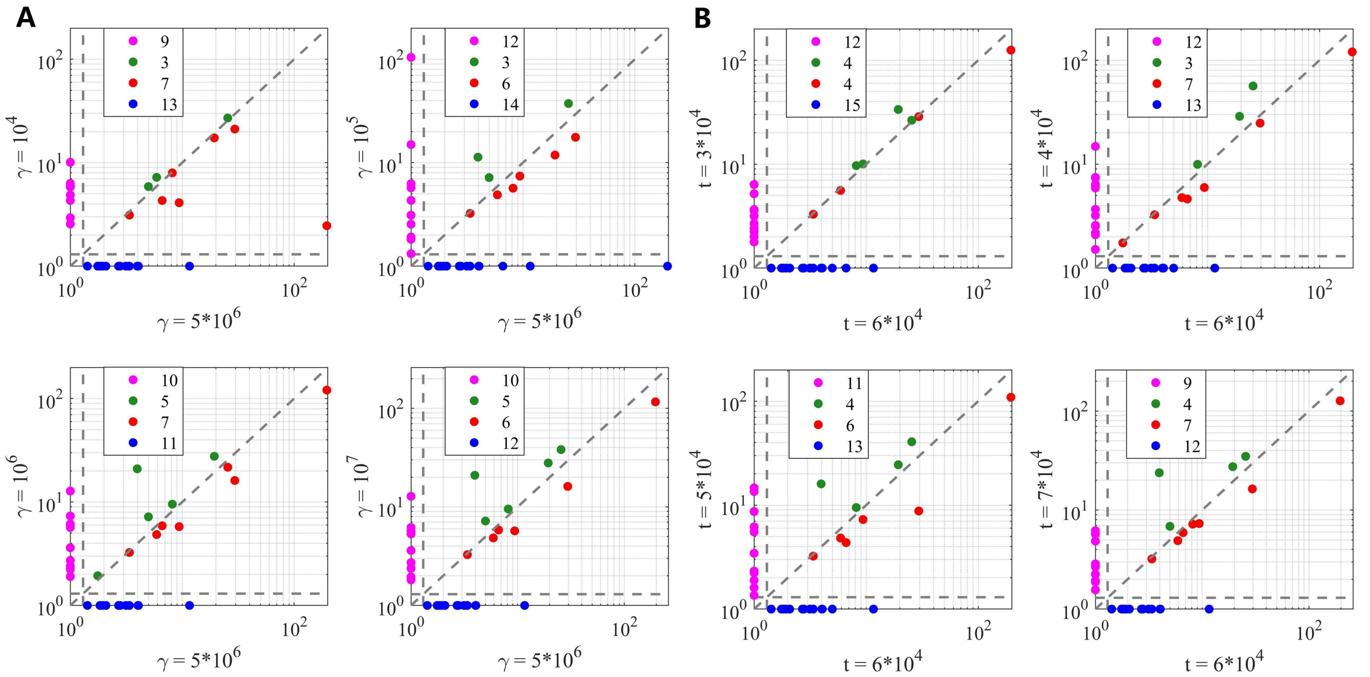

Figure S5. Results of HetFCM under different input values of γ and t on Maize dataset. **A** compares parameter setting ($\gamma = 5*10^6$) with other four ($\gamma = 10^4$, $\gamma = 10^5$, $\gamma = 10^6$ and $\gamma = 10^7$) based on enrichment analysis. **B** compares parameter setting ($t = 6*10^4$) with other four ($t = 3*10^4$, $t = 4*10^4$, $t = 5*10^4$ and $t = 7*10^4$) based on enrichment analysis.

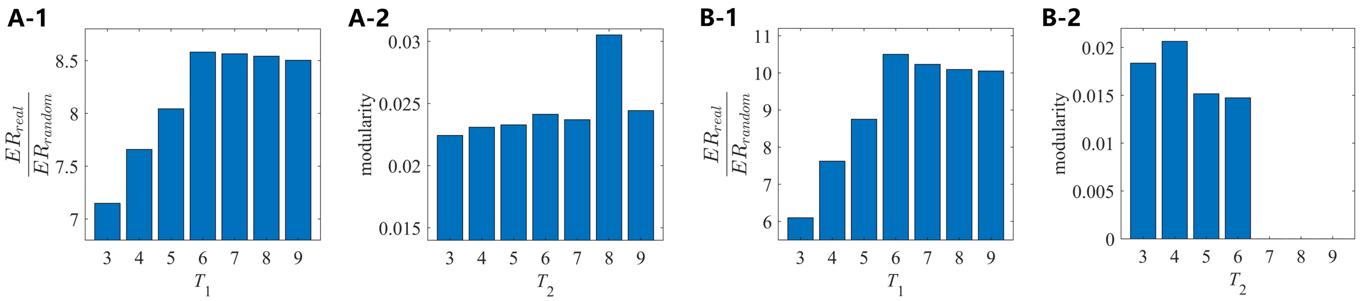

Figure S6. Results of HetFCM under different input values of T_1 and T_2. **A-1/B-1** reports the ratio between enrichment rate of detected modules and the mean of 100 random ones under different T_1 on Human/Maize dataset. **A-2/B-2** gives the modularity under different T_2 on Human/Maize dataset.

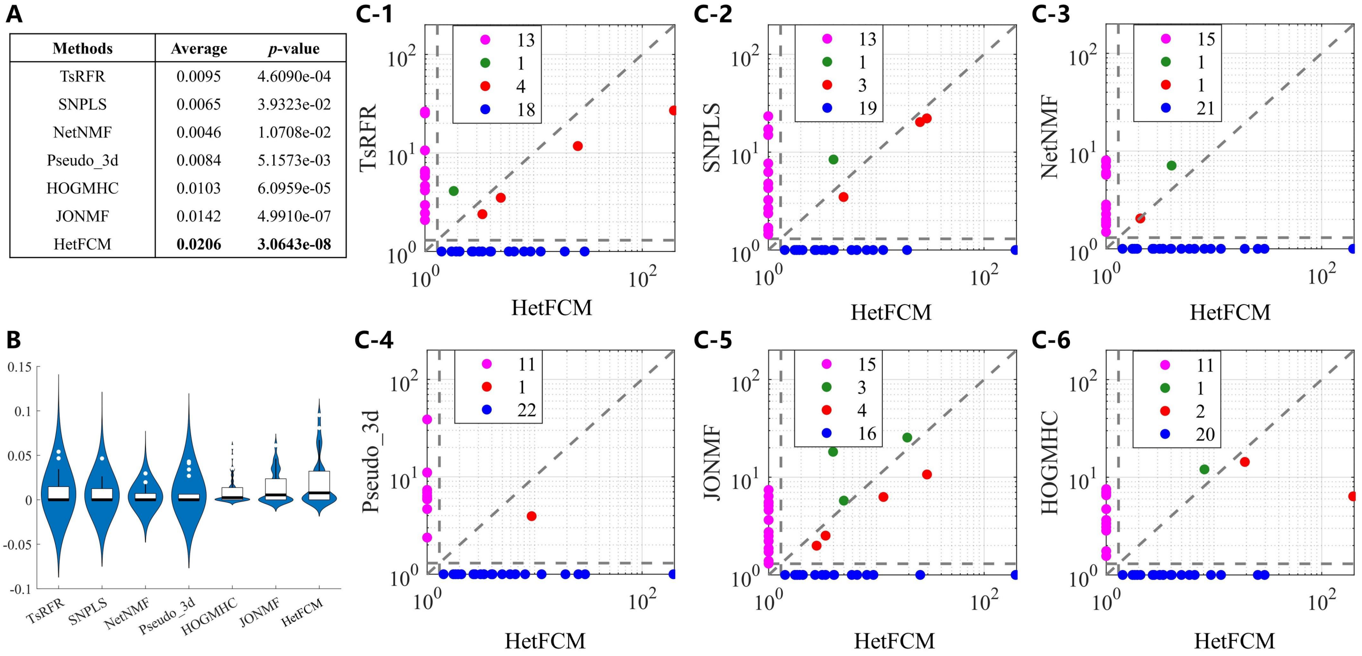

Methods	Average	*p*-value
TsRFR	0.0095	4.6090e-04
SNPLS	0.0065	3.9323e-02
NetNMF	0.0046	1.0708e-02
Pseudo_3d	0.0084	5.1573e-03
HOGMHC	0.0103	6.0959e-05
JONMF	0.0142	4.9910e-07
HetFCM	**0.0206**	**3.0643e-08**

Figure S7. Performance comparison of HetFCM and other methods at module level evaluation on Maize dataset. **A** reports the modularity of gene-miRNA co-modules detected by HetFCM and other methods. **B** shows the distribution of modularity of co-modules detected by all methods. **C** reveals the enrichment degree of GO terms. The horizontal and vertical axis represents the evaluation scores of HetFCM and other methods, respectively. The horizontal and vertical dash lines on the plot both represent the enrichment threshold (Benjamini Hochberg-corrected p-value=0.05). Magenta points denote enriched GO terms detected only by the compared method while blue ones are only by HetFCM. Both the green and red points represent GO terms simultaneously enriched by HetFCM and compared methods. Red points under the diagonal dash line mean that HetFCM has higher evaluation scores than compared methods, while green points above the diagonal dash line mean the opposite.

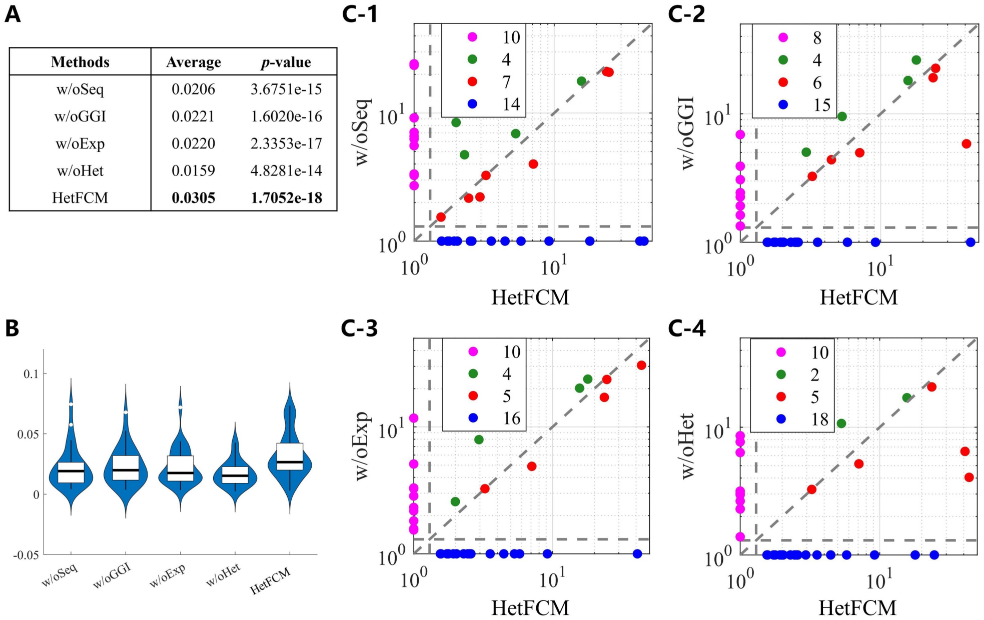

Methods	Average	*p*-value
w/oSeq	0.0206	3.6751e-15
w/oGGI	0.0221	1.6020e-16
w/oExp	0.0220	2.3353e-17
w/oHet	0.0159	4.8281e-14
HetFCM	**0.0305**	**1.7052e-18**

Figure S8. Performance comparison of HetFCM and its variants at module level evaluation on Human dataset. **A** reports the modularity of gene-miRNA co-modules detected by HetFCM and its variants. **B** shows the distribution of modularity of co-modules detected by HetFCM and its variants. **C** reveals the enrichment degree of biological processes. The horizontal and vertical dash lines on the plot both represent the enrichment threshold (Benjamini Hochberg-corrected p-value=0.05). Magenta points denote enriched biological processes detected only by the variants while blue ones are only by HetFCM. Both the green and red points represent biological processes simultaneously enriched by HetFCM and its variants. Red points under the diagonal dash line mean that HetFCM has higher evaluation scores than its variants, while green points above the diagonal dash line mean the opposite.

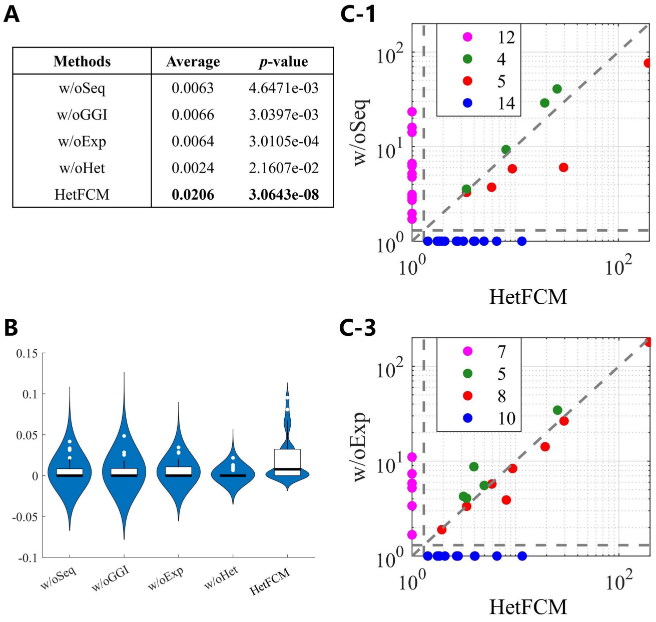

Methods	Average	*p*-value
w/oSeq	0.0063	4.6471e-03
w/oGGI	0.0066	3.0397e-03
w/oExp	0.0064	3.0105e-04
w/oHet	0.0024	2.1607e-02
HetFCM	**0.0206**	**3.0643e-08**

Figure S9. Performance comparison of HetFCM and its variants at module level evaluation on Maize dataset. **A** reports the modularity of gene-miRNA co-modules detected by HetFCM and its variants. **B** shows the distribution of modularity of co-modules detected by HetFCM and its variants. **C** reveals the enrichment degree of GO terms. The horizontal and vertical axis represents the evaluation scores of HetFCM and its variants, respectively. The horizontal and vertical dash lines on the plot both represent the enrichment threshold (Benjamini Hochberg-corrected *p*-value=0.05). Magenta points denote enriched GO terms detected only by the variants while blue ones are only by HetFCM. Both the green and red points represent GO terms simultaneously enriched by HetFCM and its variants. Red points under the diagonal dash line mean that HetFCM has higher evaluation scores than its variants, while green points above the diagonal dash line mean the opposite.

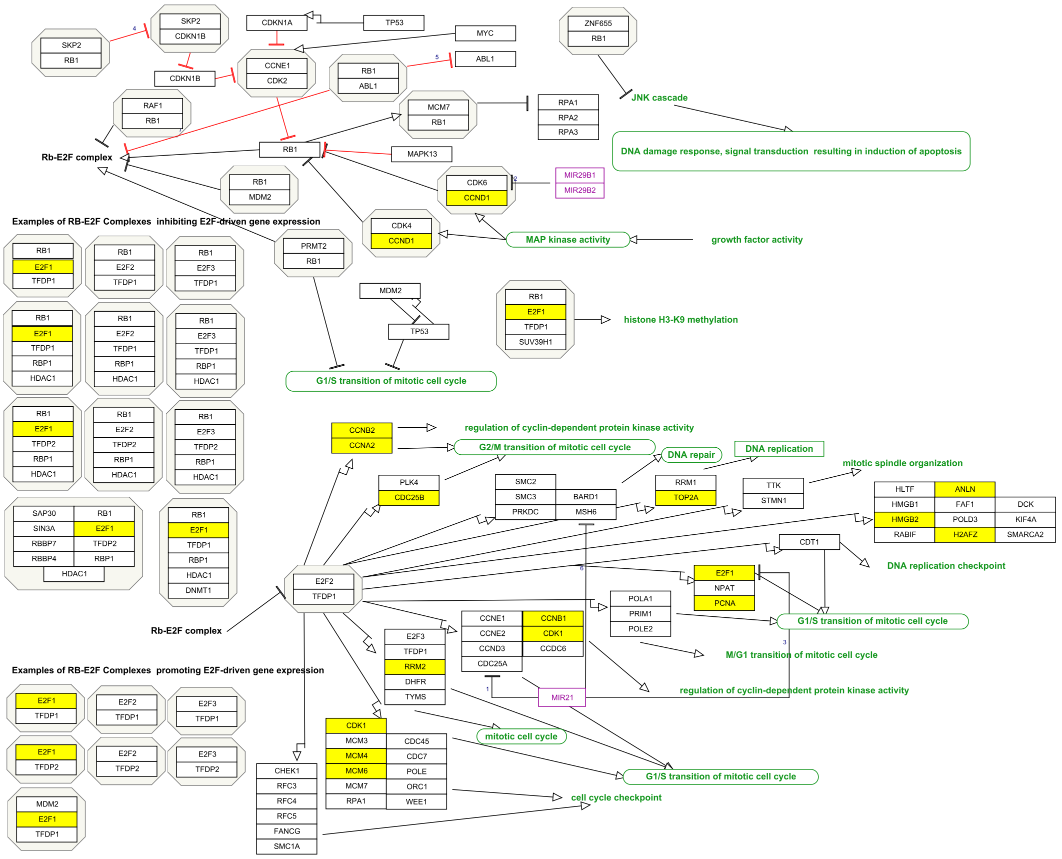

Figure S10. WikiPathway WP2446 (retinoblastoma gene in cancer) enriched by the gene layer of co-module 2. The rectangle boxes are the genes involved in this pathway, while the yellow boxes are genes within the gene layer.

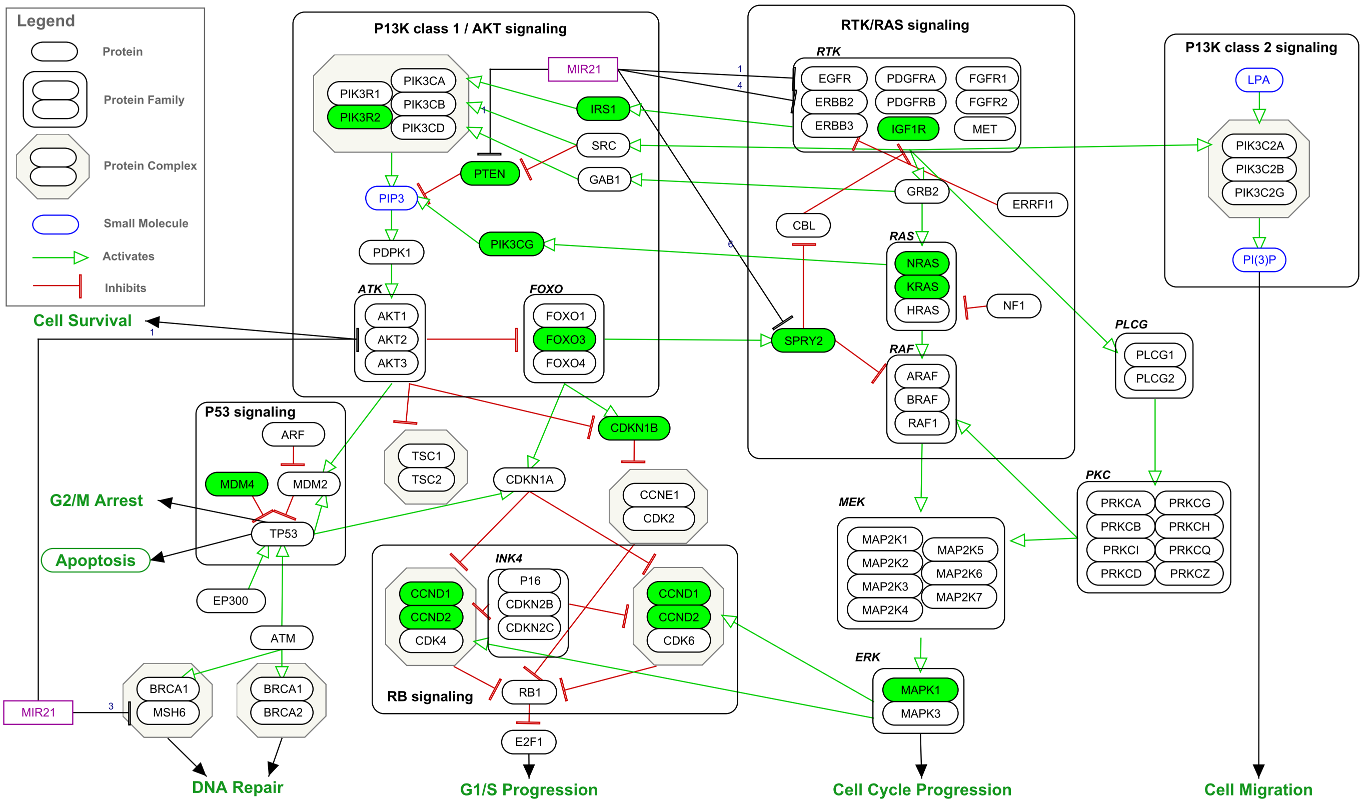

Figure S11. WikiPathway WP2261 (glioblastoma signaling pathways) enriched by the miRNA layer of co-module 14. The rectangle boxes are the genes involved in this pathway, while the green boxes are genes within the miRNA layer.

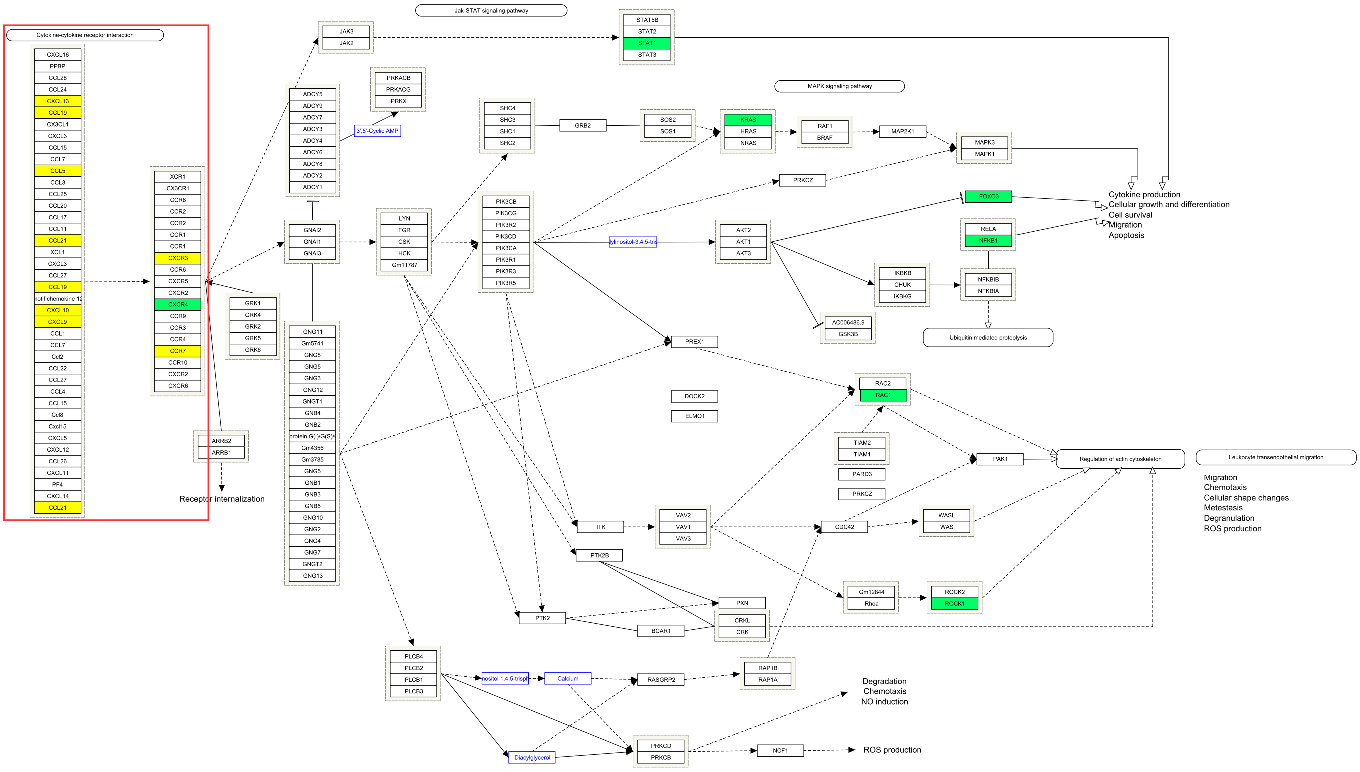

Figure S12. WikiPathway WP3929 (chemokine signaling pathway) enriched by co-module 20. The rectangle boxes are the genes involved in this pathway, while the yellow boxes are genes within the gene layer, and the green boxes are the genes corresponding to the miRNA layer.

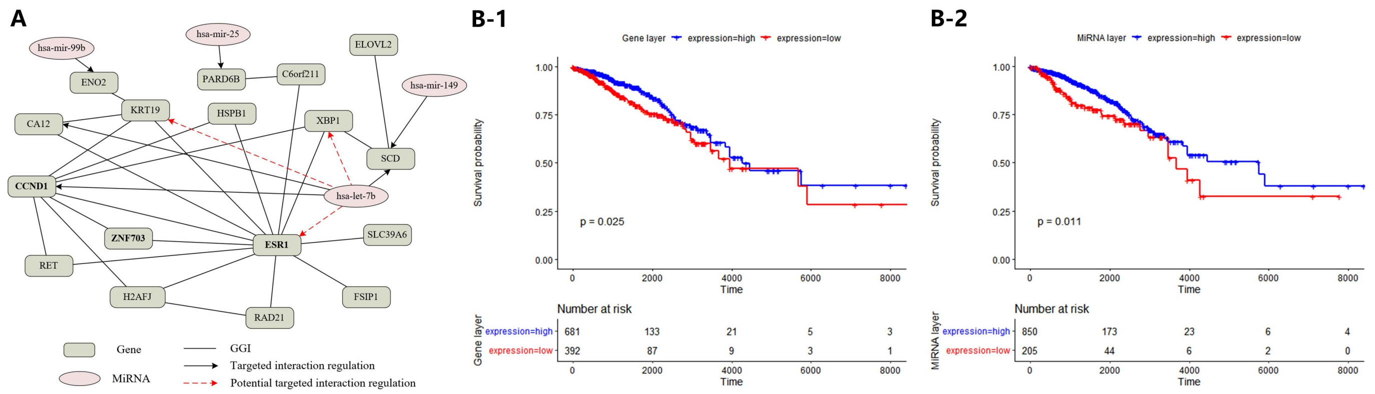

Figure S13. Information of gene-miRNA co-module 17 on Human breast cancer dataset. **A** reports interactions between molecules in co-module. **B** are Kaplan–Meier survival analysis results on gene layer and miRNA layer.

REFERENCES

1. Hsi-Yuan Huang, Yang-Chi-Dung Lin, Jing Li, Kai-Yao Huang, Sirjana Shrestha, Hsiao-Chin Hong, Yun Tang, Yi-Gang Chen, Chen-Nan Jin, Yuan Yu, et al. mirtarbase 2020: updates to the experimentally validated microrna–target interaction database. *Nucleic Acids Res.*, 48(D1):D148–D154, 2020.
2. Damian Szklarczyk, Annika L Gable, David Lyon, Alexander Junge, Stefan Wyder, Jaime Huerta-Cepas, Milan Simonovic, Nadezhda T Doncheva, John H Morris, Peer Bork, et al. String v11: protein–protein association networks with increased coverage, supporting functional discovery in genome-wide experimental datasets. *Nucleic Acids Res.*, 47(D1):D607–D613, 2019.
3. Zhonglong Guo, Zheng Kuang, Yongxin Zhao, Yang Deng, Hao He, Miaomiao Wan, Yihan Tao, Dong Wang, Jianhua Wei, Lei Li, et al. Pmiren2. 0: from data annotation to functional exploration of plant micrornas. *Nucleic Acids Res.*, 50(D1):D1475–D1482, 2022.
4. Xiangxiang Zhang, Meiyan Hong, Heping Wan, Lixia Luo, Zeen Yu, and Ruixing Guo. Identification of key genes involved in embryo development and differential oil accumulation in two contrasting maize genotypes. *Genes*, 10(12):993, 2019.
5. Max Welling and Thomas N Kipf. Semi-supervised classification with graph convolutional networks. In *International Conference on Learning Representations (ICLR)*, 2016.
6. Diederik P Kingma and Max Welling. Stochastic gradient vb and the variational auto-encoder. In *International Conference on Learning Representations (ICLR)*, 2014.
7. Wenwen Min, Juan Liu, Fei Luo, and Shihua Zhang. A two-stage method to identify joint modules from matched microrna and mrna expression data. *IEEE Trans. Nanobiosci.*, 15(4):362–370, 2016.
8. Jiazhou Chen, Hong Peng, Guoqiang Han, Hongmin Cai, and Jiulun Cai. Hogmmnc: a higher order graph matching with multiple network constraints model for gene–drug regulatory modules identification. *Bioinformatics*, 35(4):602–610, 2019.
9. Qiu Xiao, Jiawei Luo, Cheng Liang, Guanghui Li, Jie Cai, Pingjian Ding, and Ying Liu. Identifying lncrna and mrna co-expression modules from matched expression data in ovarian cancer. *IEEE/ACM Trans. Comp. Biol. Bioinf.*, 17(2):623–634, 2018.
10. John A Hartigan and Manchek A Wong. Algorithm as 136: A k-means clustering algorithm. *Journal of the royal statistical society. series c (applied statistics)*, 28(1):100–108, 1979.
11. MA Syakur, BK Khotimah, EMS Rochman, and Budi Dwi Satoto. Integration k-means clustering method and elbow method for identification of the best customer profile cluster. In *IOP conference series: materials science and engineering*, volume 336, page 012017. IOP Publishing, 2018.
12. Shihua Zhang, Qingjiao Li, Juan Liu, and Xianghong Jasmine Zhou. A novel computational framework for simultaneous integration of multiple types of genomic data to identify microrna-gene regulatory modules. *Bioinformatics*, 27(13):i401–i409, 2011.
13. David L Wheeler, Tanya Barrett, Dennis A Benson, Stephen H Bryant, Kathi Canese, Vyacheslav Chetvernin, Deanna M Church, Michael DiCuccio, Ron Edgar, Scott Federhen, et al. Database resources of the national center for biotechnology information. *Nucleic Acids Res.*, 35(suppl_1):D5–D12, 2007.
14. Tong Li, Yimin Li, Yaqi Gan, Ruotong Tian, Qihan Wu, Guang Shu, and Gang Yin. Methylation-mediated repression of mir-424/503 cluster promotes proliferation and migration of ovarian cancer cells through targeting the hub gene kif23. *Cell Cycle*, 18(14):1601–1618, 2019.
15. Wei Jian, Xiao-Chong Deng, Amik Munankarmy, Oyungerel Borkhuu, Chang-Le Ji, Xue-Hui Wang, Wen-Fang Zheng, Yun-He Yu, Xi-Qian Zhou, and Lin Fang. Kif23 promotes triple negative breast cancer through activating epithelial-mesenchymal transition. *Gland Surgery*, 10(6):1941, 2021.
16. Peter S Linsley, Janell Schelter, Julja Burchard, Miho Kibukawa, Melissa M Martin, Steven R Bartz, Jason M Johnson, Jordan M Cummins, Christopher K Raymond, Hongyue Dai, et al. Transcripts targeted by the microrna-16 family cooperatively regulate cell cycle progression. *Molecular and Cellular Biology*, 27(6):2240–2252, 2007.
17. Yuexiong Yi, Yanyan Liu, Wanrong Wu, Kejia Wu, and Wei Zhang. Reconstruction and analysis of circrna-mirna-mrna network in the pathology of cervical cancer. *Oncology Reports*, 41(4):2209–2225, 2019.
18. Walter Imagawa, Gautam K Bandyopadhyay, and Satyabrata Nandi. Regulation of mammary epithelial cell growth in mice and rats. *Endocrine Reviews*, 11(4):494–523, 1990.
19. Frederik Holst, Phillip R Stahl, Christian Ruiz, Olaf Hellwinkel, Zeenath Jehan, Marc Wendland, Annette Lebeau, Luigi Terracciano, Khawla Al-Kuraya, Fritz Jänicke, et al. Estrogen receptor alpha (esr1) gene amplification is frequent in breast cancer. *Nat. Genet.*, 39(5):655–660, 2007.
20. Somaia Elsheikh, Andrew R Green, Mohammed A Aleskandarany, Matthew Grainge, Claire E Paish, Maryou BK Lambros, Jorge S Reis-Filho, and Ian O Ellis. Ccnd1 amplification and cyclin d1 expression in breast cancer and their relation with proteomic subgroups and patient outcome. *Breast Cancer Research and Treatment*, 109(2):325–335, 2008.
21. Daniel G Holland, Angela Burleigh, Anna Git, Mae A Goldgraben, Pedro A Perez-Mancera, Suet-Feung Chin, Antonio Hurtado, Alejandra Bruna, H Raza Ali, Wendy Greenwood, et al. Znf703 is a common luminal b breast cancer oncogene that differentially regulates luminal and basal progenitors in human mammary epithelium. *EMBO Molecular Medicine*, 3(3):167–180, 2011.
22. Guoxian Yu, Keyao Wang, Carlotta Domeniconi, Maozu Guo, and Jun Wang. Isoform function prediction based on bi-random walks on a heterogeneous network. *Bioinformatics*, 36(1):303–310, 2020.
23. Esra Bozgeyik. Bioinformatic analysis and in vitro validation of let-7b and let-7c in breast cancer. *Comp. Biol. Chem.*, 84:107191, 2020.
24. SK Saha, HY Choi, BW Kim, AA Dayem, GM Yang, KS Kim, YF Yin, and SG Cho. Krt19 directly interacts with β-catenin/rac1 complex to regulate numb-dependent notch signaling pathway and breast cancer properties. *Oncogene*, 36(3):332–349, 2017.
25. Xi Chen, Dimitrios Iliopoulos, Qing Zhang, Qianzi Tang, Matthew B Greenblatt, Maria Hatziapostolou, Elgene Lim, Wai Leong Tam, Min Ni, Yiwen Chen, et al. Xbp1 promotes triple-negative breast cancer by controlling the hif1α pathway. *Nature*, 508(7494):103–107, 2014.
26. Juanjuan Kang, Qiang Tang, Jun He, Le Li, Nianling Yang, Shuiyan Yu, Mengyao Wang, Yuchen Zhang, Jiahao Lin, Tianyu Cui, et al. Rnainter

v4. 0: Rna interactome repository with redefined confidence scoring system and improved accessibility. *Nucleic Acids Res.*, 50(D1):D326–D332, 2022.
